# Investigation of metabolic crosstalk between host and pathogenic *Clostridioides difficile via* multiomics approaches

**DOI:** 10.3389/fbioe.2022.971739

**Published:** 2022-09-02

**Authors:** Ji-Eun Kwon, Sung-Hyun Jo, Won-Suk Song, Jae-Seung Lee, Hyo-Jin Jeon, Ji-Hyeon Park, Ye-Rim Kim, Ji-Hyun Baek, Min-Gyu Kim, Seo-Young Kwon, Jae-Seok Kim, Yung-Hun Yang, Yun-Gon Kim

**Affiliations:** ^1^ Department of Chemical Engineering, Soongsil University, Seoul, South Korea; ^2^ Department of Laboratory Medicine, Kangdong Sacred Heart Hospital, Hallym University College of Medicine, Seoul, South Korea; ^3^ Department of Biological Engineering, Konkuk University, Seoul, South Korea

**Keywords:** *Clostridioides difficile* infection, host-pathogen interaction, LC-MS/MS, proteomics, metabolomics, *in vitro* anaerobic-aerobic coculture model

## Abstract

*Clostridioides difficile* is a gram-positive anaerobic bacterium that causes antibiotic-associated infections in the gut. *C. difficile* infection develops in the intestine of a host with an imbalance of the intestinal microbiota and, in severe cases, can lead to toxic megacolon, intestinal perforation, and even death. Despite its severity and importance, however, the lack of a model to understand host-pathogen interactions and the lack of research results on host cell effects and response mechanisms under *C. difficile* infection remain limited. Here, we developed an *in vitro* anaerobic-aerobic *C. difficile* infection model that enables direct interaction between human gut epithelial cells and *C. difficile* through the Mimetic Intestinal Host–Microbe Interaction Coculture System. Additionally, an integrative multiomics approach was applied to investigate the biological changes and response mechanisms of host cells caused by *C. difficile* in the early stage of infection. The *C. difficile* infection model was validated through the induction of disaggregation of the actin filaments and disruption of the intestinal epithelial barrier as the toxin-mediated phenotypes following infection progression. In addition, an upregulation of stress-induced chaperones and an increase in the ubiquitin proteasomal pathway were identified in response to protein stress that occurred in the early stage of infection, and downregulation of proteins contained in the electron transfer chain and ATP synthase was observed. It has been demonstrated that host cell energy metabolism is inhibited through the glycolysis of Caco-2 cells and the reduction of metabolites belonging to the TCA cycle. Taken together, our *C. difficile* infection model suggests a new biological response pathway in the host cell induced by *C. difficile* during the early stage of infection at the molecular level under anaerobic-aerobic conditions. Therefore, this study has the potential to be applied to the development of future therapeutics through basic metabolic studies of *C. difficile* infection.

## Introduction

*Clostridioides difficile* is a gram-positive, spore-forming, toxin-producing anaerobic bacteria, an enteric pathogen that causes antibiotic-associated diarrhea and colitis (Kelly and LaMont, 1998). In general, the normal gut microbiota prevents the invasion and expansion of pathogens, but when an imbalance of the normal gut flora is caused by the use of antibiotics, *C. difficile* can colonize and proliferate (Adamu and Lawley, 2013; Leffler and Lamont, 2015). *C. difficile* infection (CDI) is one of the most frequent nosocomial infections and causes inflammatory bowel diseases ranging from mild symptoms such as diarrhea to pseudomembranous colitis and toxic megacolon in severe cases (Janka and O'Grady, 2009; Czepiel et al., 2019). The pathogenesis of CDI is a highly complex multiprocess driven by numerous bacterial virulence factors. Among them, toxins A (TcdA) and B (TcdB), which are the most representative *C. difficile* virulence factors, are most responsible for the various clinical symptoms caused by CDI (Dove et al., 1990; Gunaratnam et al., 2021). Toxins enter the host cytoplasm through receptor-mediated endocytosis and inactivate the Rho family of GTPases, which act as central regulators in various intracellular signaling pathways (Pothoulakis and Lamont, 2001; Papatheodorou et al., 2018). This leads to the reorganization of the actin cytoskeleton of the host cell and ultimately leads to apoptosis (Brito et al., 2002; Hippenstiel et al., 2002). In several previous studies, the mechanism of action of the toxins could be investigated by observing the phenotypic changes in the host caused by the toxins in *C. difficile* (Pothoulakis, 2000; Nusrat et al., 2001; Janvilisri et al., 2010; Markham et al., 2021). However, CDI pathogenesis is closely related to toxins as well as various *C. difficile* virulence factors (e.g., spore proteins, cell wall proteins, flagella proteins, etc.) (Merrigan et al., 2013; Chilton et al., 2014; Péchiné et al., 2018; Gunaratnam et al., 2021). Therefore, to understand the CDI pathogenesis mechanism more accurately, it is necessary to investigate host changes and response mechanisms in an anaerobic-aerobic model in which *C. difficile*, which produces these various virulence factors, interacts directly with the host.

Recently, several studies have been conducted to treat and understand CDI, and several *in vitro* studies using human gut epithelial cells have been reported (Shaban et al., 2018; Anonye et al., 2019). *In vitro* studies based on human gut epithelial cells offer the advantage of being suitable for analyzing direct interactions between specific microorganisms and host cells, as they can overcome genetic differences in animal models and simplify organisms with complex biological systems through *in vitro* modeling (von Martels et al., 2017). To date, *in vitro* CDI studies have mainly reported the CDI improvement effect of antimicrobial agents in an aerobic environment or the role of each risk factor for *C. difficile* in host cells (Banerjee et al., 2009; Mora-Uribe et al., 2016; Valdés-Varela et al., 2016). However, in the intestinal CDI environment, *C. difficile*, an obligate anaerobe, and intestinal epithelial cells that require oxygen coexist in the same milieu, and research using models such as the actual CDI environment remains insufficient (Pothoulakis, 2000; Janvilisri et al., 2010; Shaban et al., 2018). Moreover, the molecular-level mechanisms for physiological changes in intestinal epithelial cells induced by *C. difficile*, which may be the basis for understanding and identifying the pathogenesis of CDI, remain unclear (Anonye et al., 2019; Kim et al., 2019).

In this study, an *in vitro* CDI model was developed using the Mimetic Intestinal Host–Microbe Interaction Coculture System (MIMICS) exploited in previous research, and the metabolic crosstalk mechanism between the host and highly pathogenic *C. difficile* was investigated through integrative multiomics analysis (Song et al., 2021). After anaerobic-aerobic coculture of Caco-2 cells with *C. difficile*, the validity of the CDI model was confirmed by viability analysis and toxin concentration measurement. Then, the CDI model was comparatively analyzed according to the amount of *C. difficile* toxins accumulated from the initial (early stage of infection) 12 h to the saturated toxin concentration after 48 h (late stage of infection). Next, to investigate the physiological changes and related molecular mechanisms of Caco-2 cells during the early stage of CDI, when the expression of toxins begins, liquid chromatography combined with tandem mass spectrometry (LC–MS/MS)-based proteomic and metabolomic analysis was performed. As a result, proteins involved in tight junctions and adherens junctions of intestinal epithelial cells were significantly downregulated. In addition, acting against the accumulation of nonfunctional proteins caused by infection, stress-induced upregulation of chaperone proteins and an increase in the ubiquitin proteasomal pathway, a process of protein degradation, were observed. Last, a significant reduction in ATP, a cellular energy source, has been demonstrated, with significant downregulation of proteins involved in ATP synthase and the electron transport chain (ETC) involved in the synthesis of ATP. The results of targeted metabolomics also revealed the quantitative reduction of glycolysis- and TCA cycle-related metabolites belonging to pathways responsible for energy metabolism in intestinal epithelial cells. Consequently, this study suggests a novel biological and response mechanism of host cells through multiomics-based analysis of a model of host interaction with *C. difficile* in an anaerobic-aerobic environment. Furthermore, it is expected to be utilized as a platform technology for drug screening research in the context of CDI.

## Materials and methods

### *Clostridioides difficile* strain and culture conditions

*Clostridioides difficile* KCTC 5009 (ATCC 9689) was provided by the Korean Collection for Type Cultures (KCTC) and cultured on reinforced clostridial medium (RCM) agar (Difco, BD, NJ, United States) at 37°C in an anaerobic chamber (90% N_2_, 5% CO_2_, 5% H_2_, Coy Laboratory Products, MI, United States). A single colony of *C. difficile* was inoculated into 5 ml of glucose-supplemented medium for colon bacteria (MCB) as described by Rivière et al. (2015). It was then diluted in 30 ml of fresh MCB to an initial optical density of 0.05 at 600 nm. Optical densities were measured by UV spectrophotometry (Thermo Fisher Scientific, MA, United States). *C. difficile* was cultured until the mid-log phase and centrifuged at 4000 g for 10 min at 4°C. The bacterial cells were washed with prereduced sterile phosphate buffered saline (PBS) and diluted to a concentration of 1 × 10^8^ colony forming unit (CFU) ml^−1^ with Dulbecco’s modified Eagle’s medium (DMEM; Thermo Fisher Scientific, MA, United States) supplemented with 10% fetal bovine serum (FBS; Biowest, MA, United States). *C. difficile* was preconditioned for 30 min under anaerobic conditions before coculture with Caco-2 cells in MIMICS.

### Mammalian cell culture, media, and conditions

The human gut epithelial cell line Caco-2 was distributed from the Korean Cell Line Bank (KCLB no. 30037.1). Caco-2 cells (passages 31–33) were cultured in DMEM supplemented with 10% FBS and 1% penicillin-streptomycin (Sigma–Aldrich, MO, United States) at 37°C in a CO_2_ incubator (5% CO_2_, Thermo Fisher Scientific, MA, United States). Caco-2 cells were maintained for 7 days, and 1 × 10^6^ cells were subcultured in each MIMICS. Before coculture with *C. difficile*, Caco-2 cells of MIMICS were maintained for 2 weeks for proliferation and differentiation in a CO_2_ incubator (5% CO_2_, Bionex, Seoul, South Korea). After culturing Caco-2 cells for 2 weeks, the MIMICS was washed two times with sterile PBS and filled with antibiotic-free DMEM supplemented with 10% FBS. Then, the MIMICS was placed inside the anaerobic chamber. The medium in the host-anaerobe coculture compartment was replaced with 5 ml of prereduced DMEM supplemented with 10% FBS or preconditioned *C. difficile* inoculum. Caco-2 and *C. difficile* were cocultured for 12 h or 48 h in the anaerobic chamber before analysis.

### Assessment of cell viability of human gut epithelial cells and *Clostridioides difficile*

After coculture, the MIMICS was disassembled, and the porous membrane support upon which Caco-2 cells were cultured was isolated from the MIMICS and transferred to a 60 mm Petri dish (10060, SPL Life Sciences, Korea). The porous membrane support was carefully washed three times with 10 ml sterile PBS. Next, 5 mg/ml 3-(4,5-di methylthiazol-2-yl)-2,5-diphenyltetrazolium bromide (MTT) dissolved in PBS was diluted 10-fold with cell culture medium (MTT/cell culture medium, 1:9 v/v), and then added to the porous membrane support to check the formation of purple formazan in living cells. After 4 h of incubation at 37°C, the supernatant was removed, and the porous membrane supports were photographed. Then, the stained purple formazan crystals were solubilized in lysis buffer [dimethylformamide/water (1:1 v/v) supplemented with 20% w/v sodium dodecyl sulfate (71725, Sigma–Aldrich, MO, United States), pH 4.7] at room temperature for 2 h under dark conditions. Finally, the absorbance at 570 nm was measured by UV spectrophotometry.

Cell viability of *C. difficile* was assessed by measuring CFU after coculture for 12, 24, and 48 h under anaerobic conditions compared with inoculum. Samples harvested at each point were serially diluted and spread on reinforced clostridial medium (RCM) agar plates in an anaerobic chamber. RCM agar plates with *C. difficile* were cultured in an anaerobic chamber for 2 days, and CFU was calculated. All data were analyzed by analysis of variance (ANOVA) followed by Tukey’s honestly significant difference (Tukey’s HSD) post-hoc test using the R statistical programming environment.

### Oxygen sensing in MIMICS

Needle-type oxygen microsensors (NTH-PSt7, PreSens, Regensburg, Germany) were placed in the two lids of the MIMICS. Oxygen microsensors were installed with an oxygen meter (OXY-4 ST, PreSens, Regensburg, Germany) in the anaerobic chamber. The oxygen concentrations in the host-anaerobe coculture compartment and aerobic medium compartment were measured every 30 min for 48 h.

### Enzyme-linked immunosorbent assays

Quantification of *C. difficile* toxins was determined using an enzyme-linked immunosorbent assay (ELISA) kit (TGC-E002-1, tgcBIOMICS, Bingen, Germany). Culture supernatant was obtained by centrifugation (4,000 × g, 10 min) of the medium of the host–anaerobe coculture compartment. The amount of toxin produced was measured using an ELISA kit according to the manufacturer’s instructions. Statistical analysis was performed by ANOVA test followed by Tukey’s HSD post-hoc test using the R statistical programming environment.

### Fluorescence staining

The porous membrane supports with Caco-2 cells were washed two times with sterile PBS and fixed in 4% formaldehyde solution for 5 h at room temperature. Following washing in PBS, Caco-2 cells were incubated with 0.1% Triton-X (Sigma–Aldrich, MO, United States) in PBS for 30 min. Then, the actin cytoskeleton was stained with FITC-phalloidin (Sigma–Aldrich, MO, United States) for 30 min, and the cell nucleus was stained with 4′,6-diamidino-2-phenylindole (DAPI; Sigma–Aldrich, MO, United States) for 2 min. The cells were imaged with a Nikon Eclipse Ti inverted fluorescence microscope (Nikon, Tokyo, Japan).

### Scanning electron microscopy

For scanning electron microscopy (SEM), the porous membrane supports were prefixed in 2.5% glutaraldehyde (Sigma–Aldrich, MO, United States) for 2 h at 4°C. After washing twice with sterile PBS, the samples were postfixed in 1% osmium tetroxide solution (Sigma–Aldrich, MO, United States) at 4°C for 1 h and dehydrated with 50% and 70% ethanol (Supelco, Bellefonte, PA, United States) for 10 min each. Subsequently, samples were subjected to successive dehydration steps of 80%, 90%, 95%, and 100% ethanol, each conducted twice for 10 min each. The dehydrated samples were chemically dried by adding 100% hexamethyldisilazane (Sigma–Aldrich, MO, United States) and coated with platinum. Then, the samples were analyzed with a Gemini 300 scanning electron microscope (Carl Zeiss, Jena, Germany).

### Extraction and preparation of the proteome

Intracellular proteome samples of Caco-2 cells cocultured with *C. difficile* for 12 h were prepared by the filter-aided sample preparation (FASP) method with several modifications (Wiśniewski, 2018). The porous membrane support upon which the Caco-2 cells were cultured was washed two times with sterile PBS. Caco-2 cells were lysed with 2 ml of radioimmunoprecipitation assay buffer (RIPA buffer; Thermo Fisher Scientific, MA, United States) supplemented with 0.1% v/v protease inhibitor cocktail (Sigma–Aldrich, MO, United States), followed by scraping with a cell scraper. The lysed cell solution was sonicated with a probe sonicator (Sonics & Materials Inc., CT, United States) on ice for protein extraction. After the cell lysates were centrifuged at 4,000 g for 10 min at 4°C, the supernatant was obtained. The protein concentration was measured by a bicinchoninic acid protein assay kit (BCA assay; Thermo Fisher Scientific, MA, United States) according to the manufacturer’s instructions. For protein reduction, dithiothreitol (DTT) was added to a final concentration of 50 mM and incubated for 5 min at 95°C. Then, 100 μg of reduced proteins were transferred to 30 k filter units (Microcon; Millipore, MA, United States) with 200 μl of UA buffer (8 M urea in 0.1 M Tris-HCl, pH 8.5). The filter was centrifuged at 10,000 g for 15 min at 20°C, and this step was repeated three times. Alkylation of proteins was performed by the addition of 100 μl of 0.05 M iodoacetamide (IAM) in UA buffer and incubation in the dark for 20 min at room temperature. After centrifugation, 100 μl of UA buffer was added to the concentrate and centrifuged again. This step was repeated twice, and the filter unit was centrifuged again twice with 100 μl 0.05 M Tris-HCl, pH 8.5. Subsequently, the prepared proteins were digested by trypsin overnight, and the digests were centrifuged with 250 μl of 0.05 M Tris-HCl, pH 8.5, in a filter unit. Finally, the digests were desalted on Pierce Peptide Desalting Spin Columns (Thermo Fisher Scientific, MA, United States) according to the instructions provided by the manufacturer. Purified samples were dried with a centrifugal vacuum concentrator (Vision Scientific, Seoul, Korea) and stored at −80°C until used for analysis.

Extracellular protein extraction of *C. difficile* was performed using the host-anaerobe compartment medium. For the protein precipitation, 100% trichloroacetic acid (TCA; Thermo Fisher Scientific, MA, United States) was added to the sample to adjust the final concentration to 20% TCA. The sample was incubated at 4°C for 1 h. After centrifugation at 4,000 rpm for 20 min at 4°C, the pellets were washed two times with −20°C absolute acetone. Each sample was dried at RT for 20 min, and the protein pellets were dissolved in 8 M urea. The protein concentration was measured by BCA assay according to the instructions provided by the manufacturer. For protein reduction, 10 mM DTT was added to the samples and incubated at 55°C for 30 min. Then, 0.5 M IAM was diluted 25-fold with the samples for protein alkylation and incubated at RT in darkness for 30 min. After buffer change (sample: buffer = 1:7, v/v) with 25 mM ammonium bicarbonate buffer, trypsin proteases were spiked into the samples for digestion (protein: trypsin = 50: 1, w/w) and kept at 37°C for 19 h. The obtained peptides were purified using a Sep-Pak Plus C18 cartridge (Waters, MA, United States) according to the instructions provided by the manufacturer. Purified samples were dried with a centrifugal vacuum concentrator and stored at −80°C before analysis.

### Proteome analysis by LC–MS/MS

Proteomic analysis was performed as described previously (Jo et al., 2020; Park et al., 2022). Before the analysis, the dried samples were dissolved in solvent A (0.1% v/v formic acid in water). Proteomic analysis was performed using a nano-HPLC Ultimate 3000 RSLCnano LC system (Thermo Scientific, MA, United States). A Q Exactive Hybrid Quadrupole-Orbitrap (Thermo Scientific, MA, United States) equipped with a nanoelectrospray ionization source was used in combination with nanoHPLC. Samples were trapped in an Acclaim PepMap 100 trap column (100 μm × 2 cm, nanoViper C18, 5 μm, 100 Å, Thermo Scientific, MA, United States). Then, solvent A (98%) was used to wash the column at a flow rate of 4 μl/min for 6 min. After washing, the samples were separated at a flow rate of 350 nl/min. An Acclaim PepMap 100 capillary column (75 μm × 15 cm, nanoViper C18, 3 μm, 100 Å, Thermo Scientific, MA, United States) was used for LC separation. The LC gradient was as follows: 0 min, 2% B; 30 min, 35% B; 40 min, 90% B, 45 min, 90% B; 60 min, 5% B. Solvent A (0.1% formic acid in water) and solvent B (0.1% formic acid in acetonitrile) were used. The ion spray voltage was 2,100 eV. MS data were collected using X-caliber software. The Orbitrap analyzer scanned precursor ions with a mass range of 350–1,800 m/z and a resolution of 70,000 at m/z 200. For collision-induced dissociation (CID), up to 15 of the most abundant precursor ions were selected. The normalized collision energy was 32.

Protein identification and quantification were performed using MaxQuant software as described. Carbamidomethylation of cysteines was set as a fixed modification. Oxidation of methionine and N-terminal acetylation were set as variable modifications. The protease used for cleavage was trypsin and up to two missed cleavages were allowed. “Match between runs” option was enabled. Proteins and peptides were filtered for less than 1% FDR. Proteins were quantified by label-free quantification (LFQ) (Cox et al., 2014). Proteins were identified by searching the MS and MS/MS data of peptides against the UniProt database. Statistical analysis of LFQ data was performed by Perseus software, and the significance was determined using the adjusted *p*-value (Tyanova et al., 2016). Proteins with a fold-change of 1.5 or more and a *p*-value of 0.05 or less were defined as quantitatively significant.

### Preparation and analysis of intracellular metabolites of Caco-2 cells

To prepare intracellular metabolites, the porous membrane support that the Caco-2 cells were cultured on was washed two times with sterile PBS. Caco-2 cells on the porous membrane support were lysed with 5 ml of ice-cold 80% v/v methanol solution, followed by incubation for 4 h at −70°C. The cell lysis solution was centrifuged at 4,000 g for 10 min at 4°C. Then, the supernatant was divided into four microcentrifuge tubes and dried in a centrifugal vacuum concentrator. Before analysis by LC-MS/MS, dried metabolite samples were dissolved in 50% methanol containing 10 μg/ml l-phenylalanine-^13^C_9_ (Sigma–Aldrich, MO, United States) as internal standards. Finally, they were filtered with a syringe filter (PVDF, pore size 0.45 μm, Millipore, Billerica, United States) before analysis.

Metabolomic analysis was performed as described previously (Lee et al., 2022). Prepared samples were transferred to LC vials and analyzed by LC–MS/MS by multiple reaction monitoring (MRM) methods with the 188 internal metabolite libraries. Metabolomic analysis was performed using a 1260 Infinity Binary HPLC system (Agilent, CA, United States) combined with a 6420 Triple Quadrupole LC–MS system (Agilent, CA, United States). Injected metabolites were separated using an X-bridge amide column (4.6 × 100 mm, 3.5 μm particle size, Waters, Milford, MA, United States) with a solvent A (20 mM ammonium acetate (HPLC grade, Fisher Scientific, NJ, United States) and 20 mM ammonium hydroxide (HPLC grade, Fisher Scientific, NJ, United States) in 95:5 water: acetonitrile) and solvent B (100% acetonitrile) gradient for analysis. The flow rate was 0.4 ml/min, and the electrospray ionization voltage was 4 kV. The LC gradient was as follows: 0 min, 85% B; 5 min, 45% B; 16 min, 0% B; 24 min, 0% B; 25 min, 85% B; and 32 min, 85% B. MS peak areas were extracted using Agilent MassHunter Qualitative Analysis software. The peak area was normalized using the peak area of the internal standard. The False Discovery Rate (FDR)-corrected *p*-values were analyzed using the statistical analysis module of Metaboanalyst 5.0 (Pang et al., 2021).

### STRING network analysis of intracellular proteins of Caco-2 cells

The intracellular proteome results of Caco-2 cells cultured with *C. difficile* for 12 h were analyzed in the web-based “Search Tool for the Retrieval of Interacting Genes/Proteins” (STRING; version 11.5, string-db.org) (Szklarczyk et al., 2021). STRING is a functional proteomic tool to analyze protein interactions based on gene ontology (GO) terms. A confidence score of 0.7 for the interaction of the two proteins was considered to indicate high association. We separately identified the enriched biological processes of up- and downregulated proteins and observed the relationships between them with high confidence. The protein-protein interaction network and data were then downloaded from STRING (file format is “PNG”, tab-delimited format) (Supplementary Figures S1, S2).

### Statistical analysis

Statistical analysis was performed using the R statistical environment. Data are expressed as the means ± SD. Statistical comparison is indicated with *, **, and *** for *p* < 0.05, *p* < 0.01, and *p* < 0.001, respectively.

## Results and discussion

### Development of CDI model using the MIMICS

**FIGURE 1:**
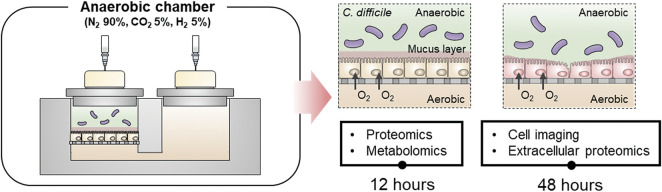
Overall scheme of the coculture process between human gut epithelial cells and *C. difficile* to analyze an integrative multiomics approach at 12 h and confirm toxin-mediated CDI at 48 h.

**FIGURE 2:**
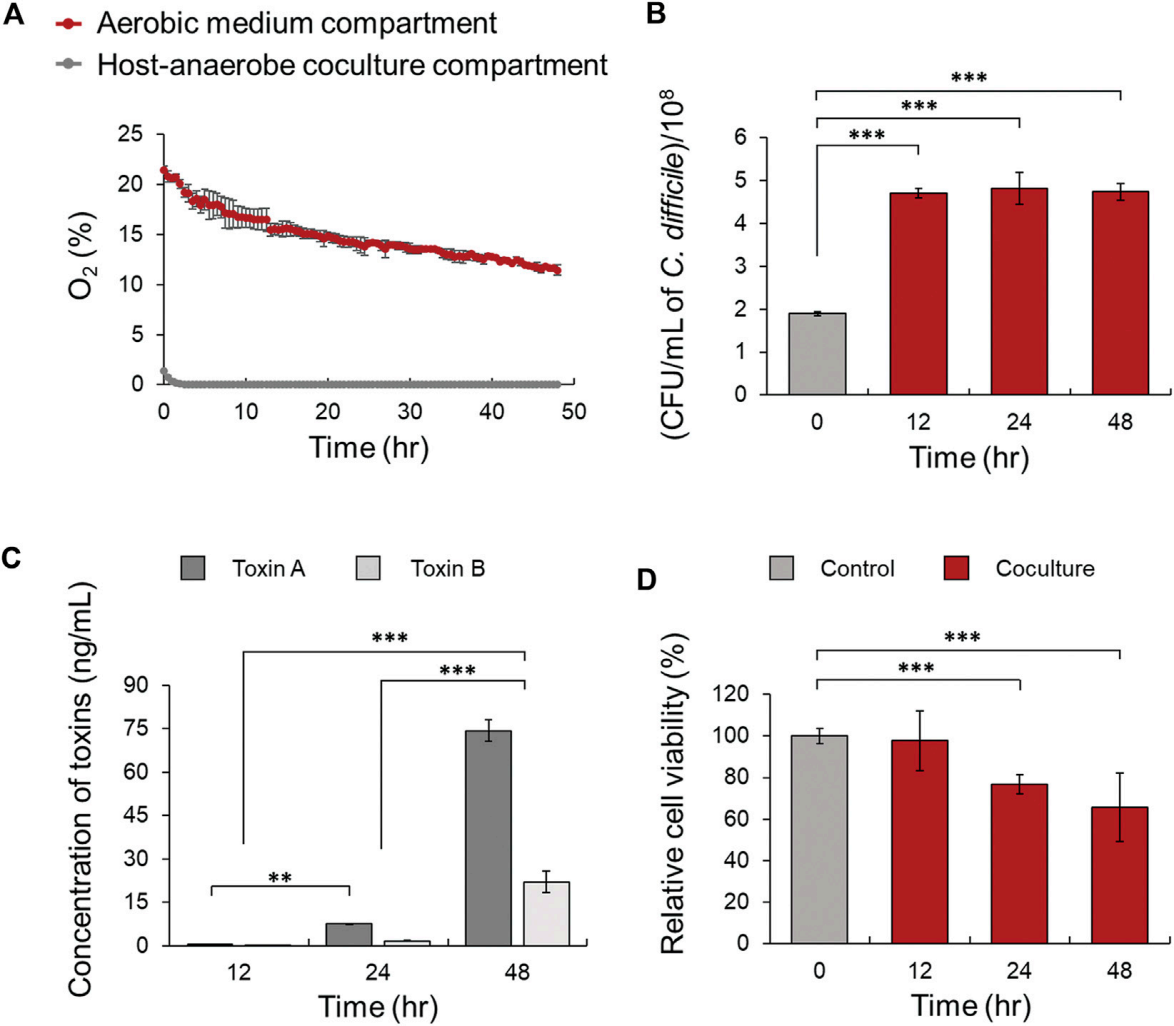
**(A)** Comparison of oxygen concentration in the host-anaerobe coculture and aerobic medium compartments in the MIMICS. **(B)** Comparison of the viability of *C. difficile* cultured with Caco-2 cells in MIMICS for 0, 12, 24, and 48 h. **(C)** Production of toxins A and B at 12, 24, and 48 h. **(D)** Comparison of the viability of Caco-2 cells according to coculture with *C. difficile* at 0, 12, 24, and 48 h compared to the control. Control means monoculture of Caco-2 cells. The error bars represent the standard deviation of triplicate samples. *p < 0.05, **p < 0.01, ***p < 0.001.

To investigate the physiological changes at the molecular level of intestinal epithelial cells due to the interaction between *C. difficile* and intestinal epithelial cells, an *in vitro* CDI model was designed using MIMICS, the Mimetic Intestinal Host–Microbe Interaction Coculture System (Figure 1) (Song et al., 2021). First, the oxygen concentration of the aerobic medium compartment over time was monitored to observe whether Caco-2 cells cultured on the porous membrane support could receive oxygen from the aerobic medium compartment below the membrane. The oxygen concentration was stably maintained for up to 48 h at approximately 10%, which is like the oxygen concentration on the serosal side of human intestinal tissue (Zeitouni et al., 2016). Moreover, the anaerobic medium compartment on the apical side was continuously maintained in an anaerobic environment with an oxygen concentration of less than 1.0% (Figure 2A). Also, there was no significant difference in the viability of Caco-2 cells for up to 48 h when the viability of MIMICS Caco-2 cells cultured in an anaerobic chamber and Caco-2 cells cultured aerobically were compared (Supplementary Figure S3, S4). The viability of the obligate anaerobe *C. difficile* was approximately 2.5 times (4.7 × 10^8^ CFU/ml) that of the inoculum (1.9 × 10^8^ CFU/ml) when cocultured for 12 h, and the level of viability was maintained similarly in cocultures for 24 and 48 h (Figure 2B). Additionally, because the concentration of toxin produced by *C. difficile* was measured according to the coculture time, TcdA and TcdB were detected at low concentrations of 0.51 and 0.25 ng/ml in the culture medium after 12 h. It was observed that both TcdA and TcdB were significantly increased with time in the culture medium after 24 and 48 h (Figure 2C). In conclusion, an *in vitro* CDI model using MIMICS demonstrated that the anaerobic-aerobic interface was stably maintained for up to 48 h and that both *C. difficile* and Caco-2 cells could remain viable in coculture for up to 48 h.

### Validation of CDI model

To investigate whether the CDI model based on anaerobic-aerobic coculture could mimic the intestinal CDI environment in the same way, we determined whether an abnormal CDI phenotype was generated due to the direct interaction between gut epithelial cells and *C. difficile*. First, the viability of Caco-2 cells treated with *C. difficile* was measured in the 12-, 24-, and 48-h CDI models. Compared with Caco-2 cells not cocultured with *C. difficile* (control), Caco-2 cells cocultured for 12 h did not show significant changes in viability. In the 24- and 48-h CDI models, the relative cell viability decreased with time to 77% and 59%, respectively (Figure 2D). In the image of MTT staining of Caco-2 cells cocultured for 48 h, desorption of gut epithelial cells and cell leakage due to monolayer damage were observed (Supplementary Figure S5). Similarly, Leslie et al. observed that disruption of human intestinal organoid (HIO) epithelium and loss of paracellular barrier function by microinjection of *C. difficile* into the lumen of HIOs. On the other hand, no similar effect was observed in the case of nontoxic *C. difficile* (Leslie et al., 2015). Based on these results, the maximum coculture time was set to 48 h, when enough *C. difficile* toxins accumulated and severe damage to the Caco-2 cell monolayer occurred. Furthermore, our CDI model was validated by confirming typical phenotypes of toxin-mediated CDI in Caco-2 cells cocultured for 48 h.

**FIGURE 3:**
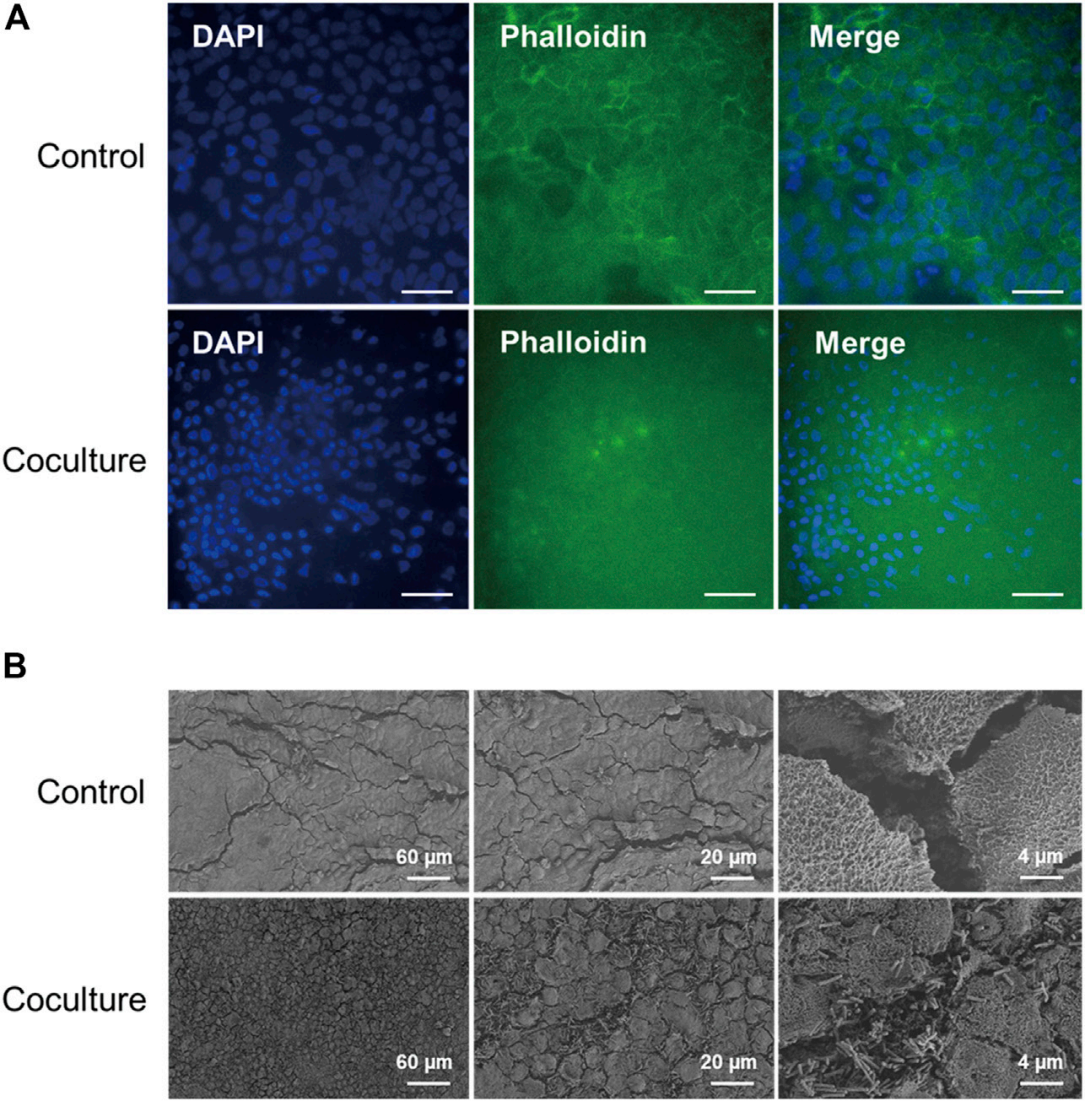
**(A)** Immunofluorescence microscopy images of Caco-2 cells in accordance with coculture with *C. difficile* at 48 h. F-actin is stained with phalloidin (green), and cell nuclei are stained with DAPI (blue). Control means monoculture of Caco-2 cells. Scale bars represent 50 µm. **(B)** Scanning electron microscopy images of Caco-2 cells in accordance with coculture with *C. difficile* at 48 h. SEM images were acquired at sequentially increased magnifications as indicated by the scale bar of each image.

First, to observe the cytoskeletal changes induced by toxins in the 48 h CDI model sample, fluorescent staining was performed on F-actin, which is responsible for cell shape and architecture. As a result, actin filaments of normal Caco-2 cells effectively maintained their morphology around the nucleus, but F-actin of Caco-2 cells cocultured with *C. difficile* for 48 h was depolymerized, and most of the previous morphology was destroyed (Figure 3A) (Anonye et al., 2019). In addition, compared to the control, the size of the cell nucleus stained during coculture with *C. difficile* was relatively small, and it could be found from the DAPI image that it exhibited irregular nuclear morphology. Since these results are like nuclear morphological changes found in apoptotic cells, it could be predicted that Caco-2 cell apoptosis was caused by virulence factors, including *C. difficile* toxins (Bianco et al., 2012). Second, to visualize the cell surface changes of the Caco-2 cell monolayer according to *C. difficile* coculture, scanning electron microscopy (SEM) analysis was performed on the 48 h CDI sample. Normal Caco-2 cells have homogeneous, flat monolayers with microvilli structures and are well differentiated in our coculture system (Figure 3B). In contrast, Caco-2 cells cocultured with *C. difficile* showed a heterogeneous nonconfluent monolayer, and microvilli disruption was determined along with the attachment of *C. difficile* with a rod shape of approximately 4 µm (Figure 3B). That is, it was observed that cell rounding occurred in the morphology of Caco-2 cells due to the toxin of *C. difficile* secreted during coculture for 48 h, like collapse of the cytoskeleton in the previous fluorescent staining (Mahida et al., 1996). Cell rounding was also observed in the Human Intestinal Enteroids (HIE) model as a response to the epithelial-toxin interaction (Engevik et al., 2020).

Finally, in the 48 h CDI model, extracellular proteome analysis of *C. difficile* was performed using LC–MS/MS to reveal whether *C. difficile* also secreted multiple virulence factors other than toxins. When the relative label-free quantitation (riBAQ) values were compared, it was determined that the S-layer protein (slpA) and flagellin (fliC) of *C. difficile* were quantitatively detected to be most abundant, at 19% and 16%, respectively (Supplementary Table S1). As virulence factors of *C. difficile*, these two proteins act as adhesins mediating adherence to the host mucosa and are key elements of the colonization step, the first step of *C. difficile* pathogenesis (Tasteyre et al., 2001; Merrigan et al., 2013). Moreover, flagellar hook-associated protein 2 (fliD), cell surface protein (cwp66), 60 kDa chaperonin (groEL) and several lipoproteins, which are known to have adhesive properties for epithelial cells *in vitro*, were also detected (Supplementary Table S1) (Chilton et al., 2014; Péchiné et al., 2018). As a result, this study showed that multiple virulence factors, including toxins of *C. difficile*, were all normally expressed in anaerobic-aerobic coculture conditions and were directly involved in the CDI pathological process by interacting with host cells. In summary, the CDI model developed in this study can simulate CDI, suggesting that it is suitable for the study of metabolic crosstalk between host and pathogenic *C. difficile* interactions.

### Proteomic changes in Caco-2 cells during the early stage of infection

**FIGURE 4:**
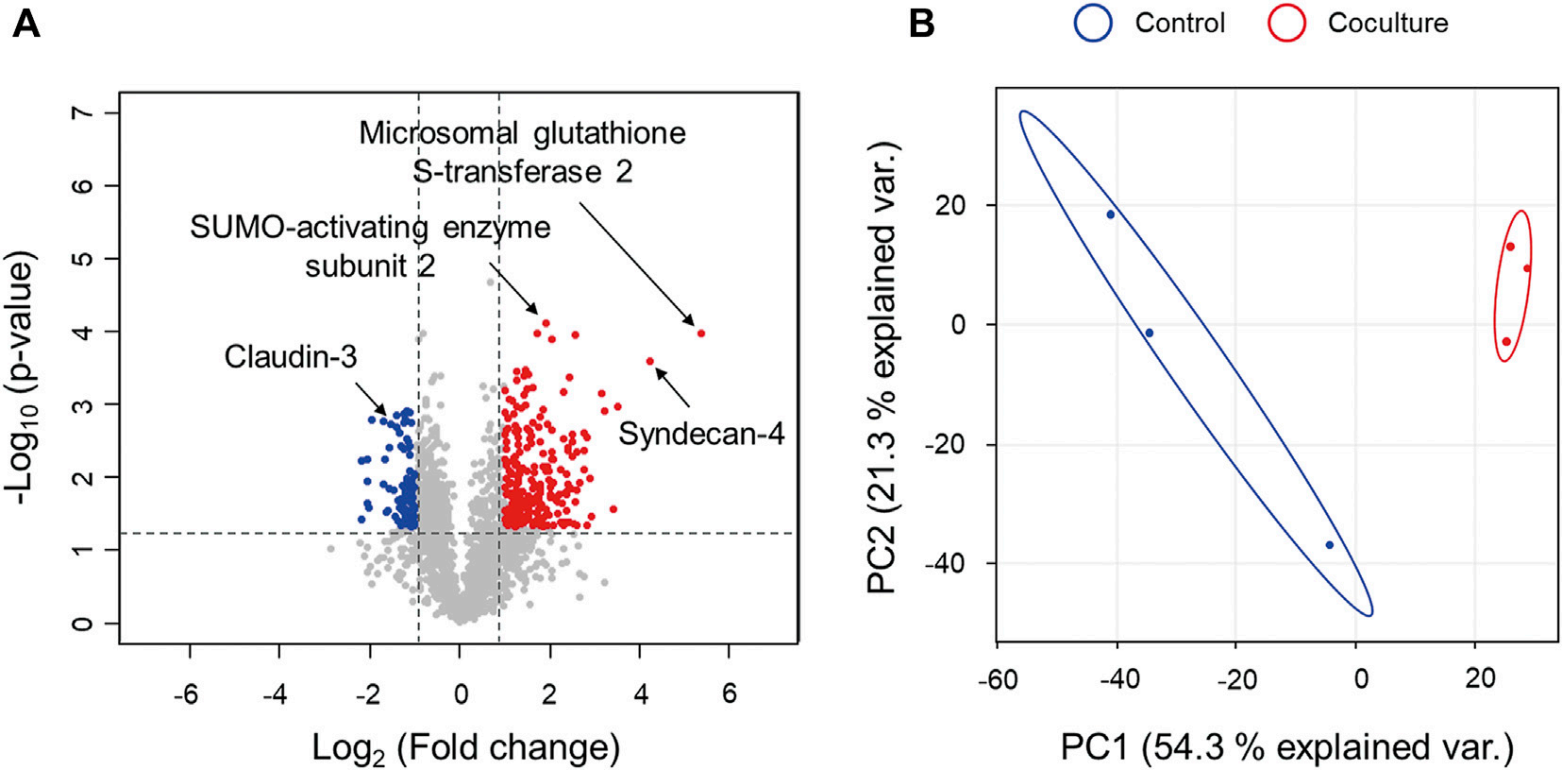
Comparison of proteomics results for Caco-2 cells in coculture with *C. difficile* at 12 h versus monoculture. **(A)** Volcano plot for comparison of proteomic differences with respect to coculture with *C. difficile* at 12 h. Volcano plots indicate the upregulated proteins of Caco-2 cells during coculture with *C. difficile* in red circles and downregulated proteins in blue circles. Indications denote the proteins discussed in this study. **(B)** Principal component analysis (PCA) plot for comparison of proteomic differences according to coculture with *C. difficile* at 12 h. Control means monoculture of Caco-2 cells. Proteomic analysis was performed in triplicate.

We selected a 12 h coculture CDI model, in which *C. difficile* proliferated sufficiently to the level of the 48 h CDI model and toxins began to be expressed, to examine the effects on the host and the host cell response mechanism in the early stage of CDI. First, intracellular proteomic analysis was performed to decipher the interactions between intestinal epithelial cells and *C. difficile*. Intracellular proteins of a total of 1,856 Caco-2 cells were detected and quantified according to the presence or absence of coculture with *C. difficile*. To classify proteins with a significant quantitative difference between the two groups, proteins with an LFQ intensity difference of more than two-fold and a *p*-value of less than 0.05 were defined as differentially expressed proteins (DEPs). As a result, the number of upregulated DEPs was 229, and the number of downregulated DEPs was 87 (Figure 4A, Supplementary Table S2). Additionally, principal component analysis (PCA) on a quantitative proteomic dataset showed that each experimental group was clustered independently, indicating a distinct change in the intracellular proteome of Caco-2 cells in the early stage of CDI (Figure 4B). We then investigated the protein–protein interaction network (PPI) analysis of 229 upregulated proteins and 87 downregulated proteins using STRING (Szklarczyk et al., 2021). First, an enrichment analysis based on the biological process categories of the Gene Ontology (GO) database was performed to categorize proteins upregulated during *C. difficile* treatment according to functional classes. It was identified that the proteins upregulated during *C. difficile* treatment were related to the protein degradation pathway, stress-induced chaperones, and glutathione-dependent antioxidant enzymes (Supplementary Figure S1). Furthermore, when downregulated proteins were identified in the same way, proteins downregulated during *C. difficile* treatment were found to be related to biological processes such as the mitochondrial ATP synthase, electron transfer chains, and mRNA translation (Supplementary Figure S2). Based on these results, we interpreted the implications of each biological pathway enriched in intestinal epithelial cells of early CDI in the pathogenesis of *C. difficile*.

### Damage to intestinal epithelial barrier function

**FIGURE 5:**
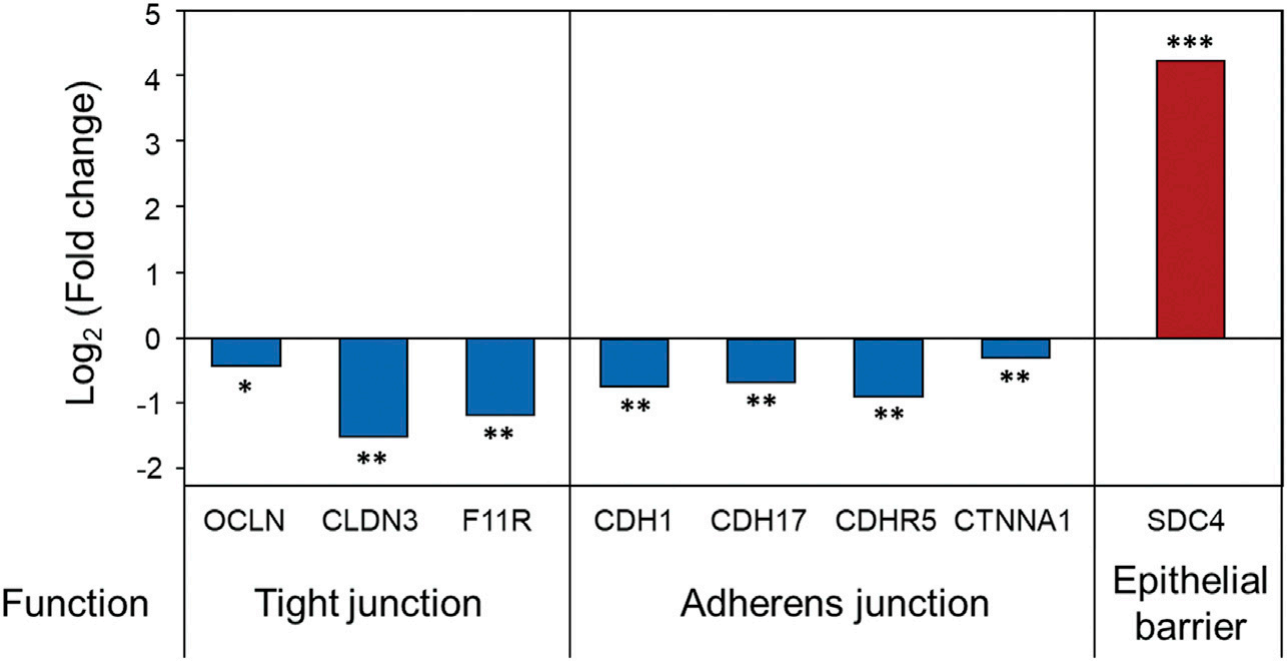
Proteomic changes in intestinal epithelial barrier function in Caco-2 cells upon coculture with *C. difficile*. Proteomic analysis was performed in triplicate. *p < 0.05, **p < 0.01, ***p < 0.001. OCLN, occludin; CLDN3, claudin-3; F11R, junctional adhesion molecule A; CDH1, cadherin-1; CDH17, cadherin-17; CDHR5, cadherin-related family member 5; CTNNA1, catenin alpha-1; SDC4, syndecan-4.

First, just as the disruption of the Caco-2 cell monolayer by toxins was confirmed in the late stage of CDI, a decrease in the barrier function of Caco-2 cells at the proteome level was also demonstrated in the early stage of CDI. Intestinal epithelial cells are connected through cell–cell junctions (i.e., tight junctions, adherens junctions, gap junctions, and the involved protein complexes are important in maintaining gut barrier function (Groschwitz and Hogan, 2009). The results of the intraproteome analysis confirmed that occludin, claudin-3, and junctional adhesion molecule A, which are tight junction proteins, were significantly downregulated by 0.72-, 0.34-, and 0.44-fold, respectively (Figure 5). In addition, cadherin-1, cadherin-17, cadherin-related family member 5, and catenin alpha-1, which are involved in adherens junctions, were all significantly downregulated (Figure 5). Based on these results, it was found that damage to the barrier of Caco-2 cells started to occur even due to virulence factors, including low concentrations of toxins expressed in the early stage of CDI. A greater quantitative decrease in tight junction proteins was observed compared to adherens junctions (Figure 5). These results suggest that, compared to the adherens junctions present in the basolateral membrane of Caco-2 cells, tight junctions close to the apical membrane were first affected by virulence factors in the early stage of CDI (Campbell et al., 2017). Interestingly, the intraproteomic results showed a significant upregulation of a protein called syndecan-4 (SDC4), which can play a role in the response to cell–cell junction damage (Figure 5). SDC4 is the second-most upregulated (19-fold) protein upon treatment with *C. difficile* and is one of the predominantly expressed syndecan proteoglycan families in the colonic epithelium (Woods, 2001). Fröhling, Mareike, et al. reported that when tight junctions are damaged in colitis-induced mice, SDC4 is upregulated for recovery, which helps maintain and regenerate intestinal epithelial integrity (Fröhling et al., 2018). The following results demonstrated that our CDI model contributed to the sequential disruption of tight junctions and adherens junctions in the early stages of infection and upregulation of SDC4 in response to tight junction damage.

### Protein refolding by stress-induced chaperones in Caco-2 cells

**FIGURE 6:**
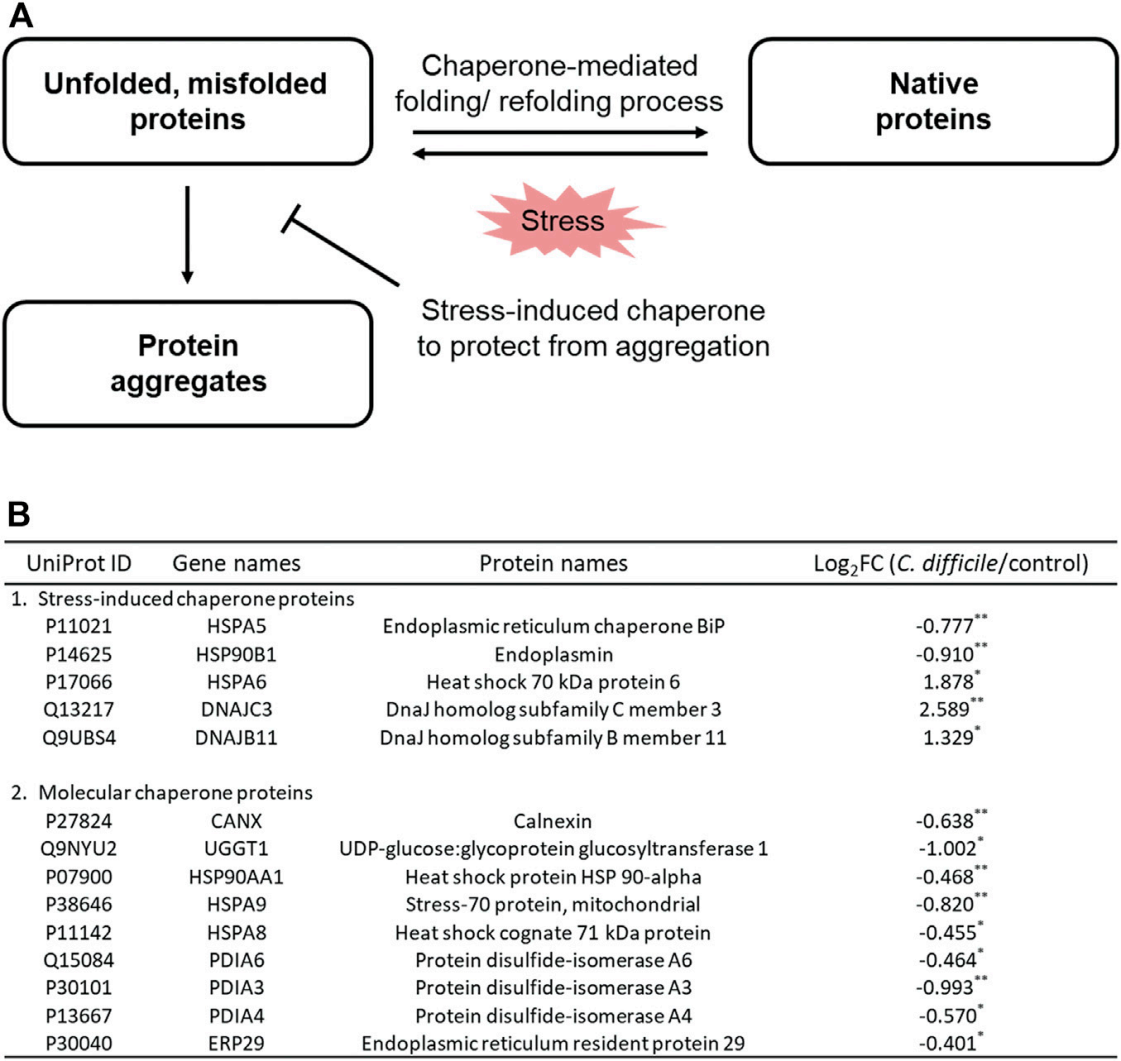
**(A)** Mechanisms of protein folding and refolding by chaperones in response to *C. difficile*-induced cellular stress. **(B)** Table of proteins belonging to the cellular chaperone family of Caco-2 cells cultured with *C. difficile*. Proteomic analysis was performed in triplicate. *p < 0.05, **p < 0.01, ***p < 0.001.

In the GO enrichment analysis of DEPs, proteins related to intracellular protein processing were enriched. Several stress factors, such as pathogen infection, can contribute to the accumulation of unfolded or misfolded proteins in the endoplasmic reticulum (ER) (Celli and Tsolis, 2015; Choi and Song, 2020). When the folding capacity of the ER is exceeded due to the excessive accumulation of these nonfunctional proteins, ER stress in the cell may occur, and the apoptosis pathway may be induced (Szegezdi et al., 2006; Jang, 2018). In response, cells attempt to maintain ER protein homeostasis through mechanisms such as refolding and repair or removal of these proteins (Jang, 2018). ER chaperone proteins induce conformational folding and/or assembly of nonfunctional proteins, thereby converting them back into functional proteins (Figure 6A) (Szegezdi et al., 2006; Celli and Tsolis, 2015). Based on the results of intraproteome analysis, it was confirmed that most chaperones in intestinal epithelial cells, including chaperone BiP (GRP78) and endoplasmin (GRP94), were downregulated by CDI, but some chaperones were interestingly significantly increased (Figure 6B). Heat shock 70 kDa protein 6 (HSP70B′), a stress-induced molecular chaperone, was increased by 3.7-fold (Figure 6B). Moreover, dnaJ homolog subfamily C member 3 and dnaJ homolog subfamily B member 11, which are co-chaperones assisting chaperones, were also significantly upregulated by 6.0 and 2.5-fold, respectively (Figure 6B). Three proteins are expressed at low levels or not expressed under normal conditions, but their overexpression is induced after severe stress, mediating the refolding or degradation of nonfunctional proteins caused by stress and preventing harmful protein aggregation (Daugaard et al., 2007; Sterrenberg et al., 2011). This result suggests that in the situation where the accumulation of unfolded or misfolded proteins is caused by multiple virulence factors from *C. difficile*, Caco-2 cells upregulate the intracellular stress-induced chaperone complex to prevent and recover from irreversible aggregation of proteins.

### Maintenance of protein homeostasis during the early stage of CDI

**FIGURE 7:**
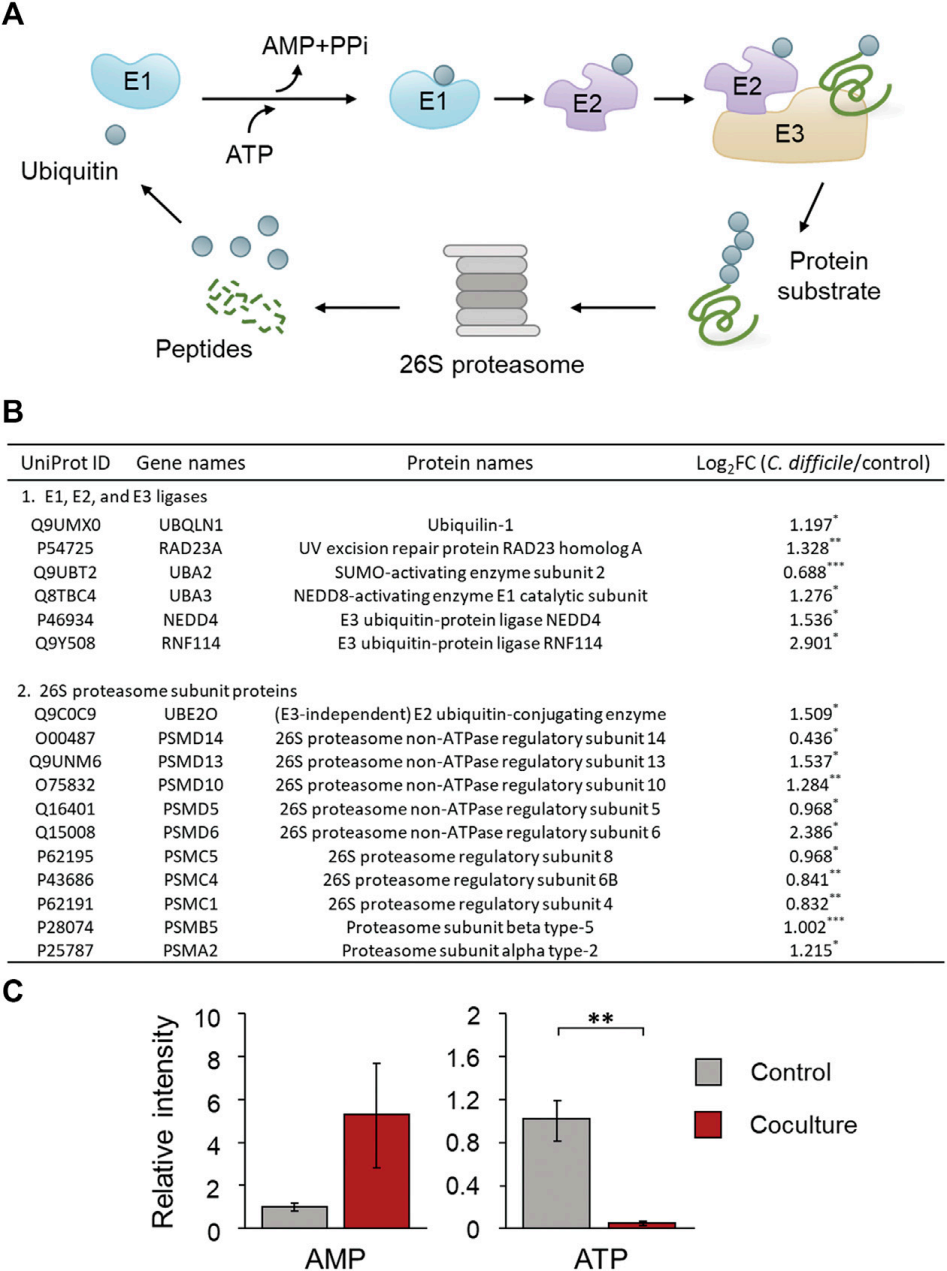
**(A)** Mechanism of the ubiquitin proteasome pathway (UPP) involved in proteolysis. Ubiquitin is conjugated to a protein substrate by three enzymes: E1, E2, and E3 ligases (Kocaturk and Gozuacik, 2018). This process is repeated until a chain of ubiquitin is formed. The ubiquitin chain is recognized by the 26S proteasome, and after the ubiquitin is removed, the protein substrate is digested to peptides. **(B)** Table of proteins belonging to the UPP of Caco-2 cells cultured with *C. difficile*. **(C)** Comparison of the production of AMP and ATP in Caco-2 cells cultured with *C. difficile*. The error bars represent the standard deviation. Metabolomic analysis was performed in quadruplicate. Proteomic analysis was performed in triplicate. *p < 0.05, **p < 0.01, ***p < 0.001.

When nonfunctional protein formation exceeds the repair capacity of the ER, abnormal cellular status is induced by cytotoxicity (Jang, 2018). Therefore, cells activate the ubiquitin proteasome pathway (UPP), an ATP/ubiquitin-dependent process, to remove these proteins and maintain cellular protein homeostasis (Lecker et al., 2006; Jang, 2018). GO enrichment analysis of upregulated DEPs confirmed that the enriched protein catabolic process involved a UPP-related protein, which may have been activated by the accumulation of abnormal proteins in Caco-2 cells, as discussed in the previous section. First, UPP is performed by the 26S proteasome, a large multiprotein complex, and starts with target protein recognition by a small peptide called ubiquitin (Lecker et al., 2006). Ubiquitin is tagged to a target protein through polyubiquitination by three enzymes: ubiquitin-activating enzyme (E1), ubiquitin-conjugating protein (E2), and ubiquitin-protein ligase (E3) (Figure 7A) (Ciechanover, 1998). Intraproteomic analysis results showed that SUMO-activating enzyme subunit 2 and NEDD8-activating enzyme E1 catalytic subunit belonging to E1 were upregulated by 1.6 and 2.4-fold, respectively. In addition, the E3 ubiquitin-protein ligase NEDD4, E3 ubiquitin-protein ligase RNF114, and E3 ubiquitin-protein ligase UBR4, which are the key enzymes for polyubiquitination, were significantly upregulated. The (E3-independent) E2 ubiquitin-conjugating enzyme with both E2 and E3 activities also showed significant upregulation (Figure 7B). These results suggest that polyubiquitination is increased in *C. difficile*-infected Caco-2 cells, thereby activating a process that degrades abnormal proteins caused by CDI. Since polyubiquitination is an ATP-dependent enzymatic reaction, when E1 activates ubiquitin, it consumes ATP and releases AMP and PPi (Figure 7A) (Ciechanover, 1998). It was demonstrated that the relative intensity of ATP significantly decreased by 0.034 times and AMP increased by 5.3 times in intestinal epithelial cells of the early stage of the CDI model based on targeted metabolomics results (Figure 7C). That is, it was considered that the consumption of ATP demanded by activating polyubiquitination in intestinal epithelial cells during the early stage of CDI contributed to the low intracellular ATP level and high intracellular AMP level. In the proteomic analysis results, several proteins belonging to the 20S core particle and 19S regulatory particle constituting the 26S proteasome were also significantly upregulated (Figure 7B). Additionally, ubiquilin-1 and the UV excision repair protein RAD23 homolog A, which are responsible for the link between the polyubiquitin chain of the target protein and the 26S proteasome subunit, were also upregulated (Figure 7B). These results indicate that 26S proteasome assembly is induced in Caco-2 cells by *C. difficile* and that proteasomal degradation of polyubiquitinated protein is activated simultaneously. In conclusion, in the early stage of CDI, intestinal epithelial cells attempt to maintain intracellular protein homeostasis by degrading abnormal proteins generated by the inhibition of chaperone-mediated protein processing through UPP.

Next, the translation process during protein synthesis, which is the opposite process of protein degradation, was enriched in the downregulated DEP GO analysis. According to several previous studies, when proteotoxic stress occurs in cells, upregulation of the proteasome and autophagy, which are proteolytic processes, and reduction of the ribosome, a component that controls protein synthesis, are induced in response (Warner, 1999; Zeiser et al., 2013; Guerra-Moreno et al., 2015). In our proteomic results, a few proteins included in the 60S large subunit and 40S small subunit of the ribosome were all significantly downregulated (Supplementary Table S3). That is, intestinal epithelial cells limit the generation of newly synthesized misfolded and unfolded proteins due to ER chaperone inhibition through the downregulation of ribosomal proteins. Based on these results, intestinal epithelial cells, in response to proteotoxic stress occurring in the early stage of CDI, not only activate UPP to remove abnormal proteins in cells but also restrict new protein synthesis. Ultimately, this suggests that Caco-2 cells seek to alleviate proteotoxic stress that can induce intracellular cytotoxicity.

### Mitochondrial dysfunction and upregulation of the antioxidant system

In the PPI analysis of a few downregulated DEPs, the GO term mitochondrial ATP synthesis coupled electron transport was enriched. First, from the proteomic analysis results, the subunits constituting the mitochondrial ATP synthase that directly synthesizes ATP were quantitatively and significantly reduced, and several proteins included in respiratory complexes I, II, III, and IV of the electron transport chain (ETC) were downregulated together (Table 1). Downregulation of these proteins first reduces the proton motive force in the mitochondrial matrix and eventually leads to a decrease in the amount of ATP synthesis. As mentioned earlier, it was revealed that the intracellular ATP level of Caco-2 cells was decreased in the early stage of CDI, and downregulation of ETC and ATP synthase-related proteins contributed to the low level of ATP (Figure 7C). Mitochondria can be easily compromised by several pathogenic bacterial effectors, such as toxins, which enter cells during bacterial infection (Lobet et al., 2015). TcdA, which corresponds to the endotoxin of pathogenic *C. difficile*, is also localized to the mitochondria of Chinese hamster ovary cells, induces mitochondrial damage, and may cause ATP depletion along with loss of mitochondrial membrane potential (He et al., 2000). Similarly, in the report by Mahida, Y. R., et al*.*, it was shown that the enzyme activities of mitochondrial dehydrogenases in Caco-2 cells were decreased by TcdA treatment (Mahida et al., 1996). Based on these results, it is demonstrated that TcdA introduced into the cell during the early stage of CDI causes a loss of mitochondrial membrane potential of intestinal epithelial cells through a quantitative decrease in protein along with a decrease in enzyme activity throughout the ETC. In addition, it was found that the intestinal epithelial cells could not replenish the ATP used for the stress response due to the inhibition of ATP synthesis due to the decrease in ATP synthase, and thus ATP was gradually depleted within the cells.

**TABLE 1:** List of identified proteins involved in the mitochondrial electron transfer chain. Proteomic analysis was performed in triplicate.

**UniProt ID**	**Gene names**	**Protein names**	**Log_2_FC (*C. difficile*/control)**
1 Mitochondrial ATP synthase			
O75964	ATP5MG	ATP synthase subunit g	−0.978*
P56385	ATP5ME	ATP synthase subunit e	−1.144*
P00846	ATP6	ATP synthase membrane subunit 6	−1.389*
P18859	ATP5PF	ATP synthase-coupling factor 6	−0.836**
P06576	ATP5F1B	ATP synthase F1 subunit beta	−0.807*
P25705	ATP5F1A	ATP synthase F1 subunit alpha	−0.734*
P48047	ATP5O	ATP synthase subunit O	−1.122**
2 Electron transport chain			
O00217	NDUFS8	NADH dehydrogenase [ubiquinone] iron-sulfur protein 8	−0.940*
Q16718	NDUFA5	NADH dehydrogenase [ubiquinone] 1 alpha subcomplex subunit 5	−1.034*
Q9Y6M9	NDUFB9	NADH dehydrogenase [ubiquinone] 1 beta subcomplex subunit 9	−1.222*
O75251	NDUFS7	NADH dehydrogenase [ubiquinone] iron-sulfur protein 7	−2.225*
P28331	NDUFS1	NADH-ubiquinone oxidoreductase 75 kDa subunit 1	−0.823*
Q99643	SDHC	Succinate dehydrogenase cytochrome b560 subunit	−1.651*
P14927	QCR7	Cytochrome b-c1 complex subunit 7	−0.862*
P22695	QCR2	Cytochrome b-c1 complex subunit 2	−1.028*
P10606	COX5B	Cytochrome c oxidase subunit 5B	−1.107*
P13073	COX4I1	Cytochrome c oxidase subunit 4 isoform 1	−0.918**
P14406	COX7A2	Cytochrome c oxidase subunit	−0.928*
P15954	COX7C	Cytochrome c oxidase subunit 7C	−1.112*
P14854	COX6B1	Cytochrome c oxidase subunit 6B1	−1.129**
Q9Y2R0	COA3	Cytochrome c oxidase assembly factor 3	−1.039*

*p < 0.05, **p < 0.01, ***p < 0.001.

**FIGURE 8:**
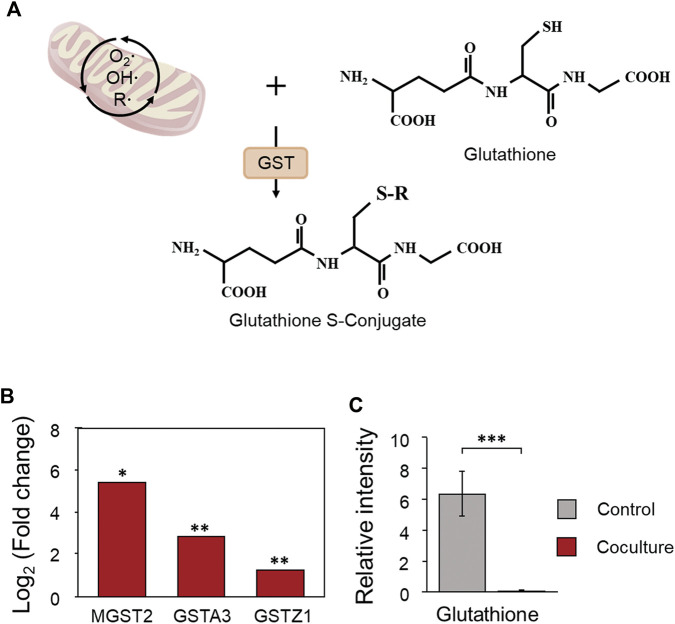
**(A)** The antioxidant role of glutathione S-transferase in Caco-2 cells during coculture with *C. difficile*. **(B)** Proteomic changes in glutathione S-transferases in Caco-2 cells during coculture with *C. difficile*. Proteomic analysis was performed in triplicate. **(C)** Comparison of the production of glutathione in Caco-2 cells cultured with *C. difficile*. The error bars represent the standard deviation. Metabolomic analysis was performed in quadruplicate. GST, glutathione S-transferase; MGST2, microsomal glutathione S-transferase 2; GSTA3, glutathione S-transferase 3; GSTZ1, glutathione S-transferase Zeta 1. *p < 0.05, **p < 0.01, ***p < 0.001.

According to previous studies, mitochondrial dysfunction of host cells caused by toxins of *C. difficile* can cause the generation of reactive oxygen species (ROS) and exert a cytotoxic effect on cells. Mitochondria are major cellular sites where peroxides are produced during ROS generation (Green and Reed, 1998; He et al., 2002; Matarrese et al., 2007). We determined the increase in the antioxidant system that protects cells from oxidative stress caused by the generation of ROS from the results of proteomics analysis (Supplementary Table S2). The cellular antioxidant system is largely composed of nonenzymatic elements such as vitamins A, C, and E and enzymatic elements such as catalase (CAT), glutathione peroxidase (GPX), and glutathione S-transferase (GST) (Hayes et al., 2005; Hellou et al., 2012). Enzymatic factors are divided into extracellular or intracellular antioxidant enzymes depending on where the defense against ROS proceeds, and among them, GST is an intracellular antioxidant enzyme that functions to detoxify or remove oxidized macromolecules by conjugating with glutathione (Figure 8A) (Hayes et al., 2005; Hellou et al., 2012). According to the proteomic analysis results, microsomal glutathione S-transferase 2 belonging to GST showed the greatest increase of 41-fold, and glutathione S-transferase A3 and glutathione S-transferase Zeta 1 were upregulated by 7.0 and 2.4-fold, respectively (Figure 8B). Microsomal glutathione S-transferase 2, which showed the greatest increase, is an enzyme with glutathione-dependent peroxidase activity, especially for lipid peroxides (Ahmad et al., 2013). Like this result, in the metabolomic analysis results, it was demonstrated that the relative intensity of glutathione consumed as a substrate for GST decreased by 0.008-fold following the activation of the antioxidant system, showing the most significant decrease (Figure 8C). In response to an increase in peroxides in intestinal epithelial cells during the early stages of CDI, these findings suggest that oxidative stress is alleviated through significant upregulation of ROS metabolizing enzymes and consumption of the antioxidant glutathione.

### Metabolomic changes in Caco-2 cells during the early stage of CDI

**FIGURE 9:**
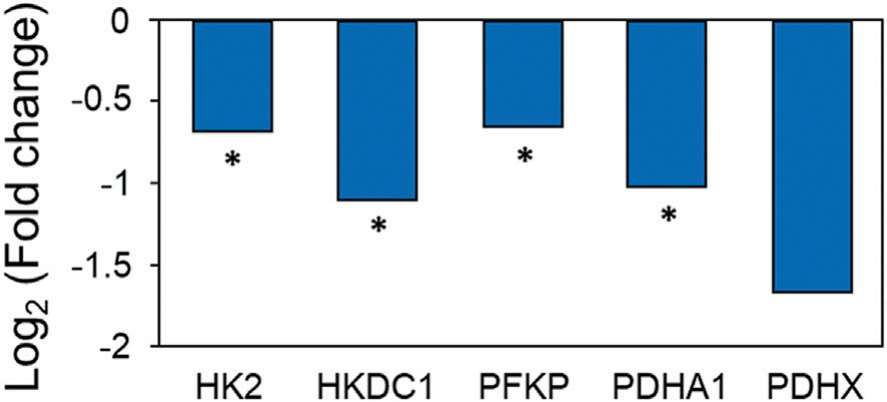
Proteomic changes in energy metabolism in Caco-2 cells upon coculture with *C. difficile*. Proteomic analysis was performed in triplicate. HK2: hexokinase 2, HKDC1, hexokinase domain containing 1; PFKP, phosphofructokinase; PDHA1, pyruvate dehydrogenase E1 component subunit alpha; PDHX, pyruvate dehydrogenase protein X component. *p < 0.05, **p < 0.01, ***p < 0.001.

Based on LC–MS/MS-based metabolite profiling, quantitative changes in intracellular metabolites of Caco-2 cells generated in the early stage of the CDI model were identified. As a result of targeted metabolomics, a total of 165 metabolites were detected: 35 metabolites were significantly upregulated [log_2_ (fold-change) >1, *p* < 0.05], and 25 metabolites were downregulated [log_2_ (fold-change) <−1, *p* < 0.05] when *C. difficile* was treated (Supplementary Figure S6A, Supplementary Table S4). Additionally, principal component analysis (PCA) of the metabolomic dataset showed that each experimental group was clustered independently, which means that there was a distinct change in the intracellular metabolites of Caco-2 cells during the early stage of CDI (Supplementary Figure S6B). First, it was confirmed that glutamate, cysteine, and ATP, which are intermediate metabolites belonging to the glutathione biosynthesis pathway, decreased by 0.38, 0.44, and 0.025 times, respectively, and the product glutathione was also significantly decreased by 0.008 times (Table 2). As discussed above, since the glutathione-based antioxidant system is activated, Caco-2 cells not only consume glutathione rapidly but also deplete metabolites used for glutathione biosynthesis. Thus, if the oxidative damage caused by *C. difficile* continues, it can be inferred that the glutathione-based antioxidant system of Caco-2 cells will not proceed normally and will ultimately cause severe damage to the cells due to ROS. Second, quantitative changes in metabolites related to glycolysis and the TCA cycle, which are the major energy metabolism pathways of Caco-2 cells, were identified. Glucose belonging to glycolysis was significantly reduced by 0.40 times, d-glucose-6-phosphate by 0.077 times, and d-fructose-6-phosphate by 0.071 times. In the TCA cycle, citrate was significantly reduced by 0.15 times, and malate was reduced by 0.22 times (Table 2). Although not significant, oxaloacetate and pyruvate were increased by 4.0 and 1.78-fold, respectively, and as a result, NADH and ATP, the main products of glycolysis and the TCA cycle, were significantly decreased by 0.25 and 0.025 times, respectively (Table 2). Intraproteomic results revealed a significant decrease in hexokinase 2, hexokinase domain containing 1, and phosphofructokinase, an enzyme belonging to glycolysis (Figure 9). The decrease in both enzymes and the decrease in d-glucose-6-phosphate and d-fructose-6-phosphate, which are products of the two enzymatic reactions, show that glycolysis is downregulated in the early stage of CDI (Table 2). The proteomic analysis confirmed that pyruvate dehydrogenase E1 component subunit alpha and pyruvate dehydrogenase protein X component, which are enzymes that generate acetyl-CoA from pyruvate, were significantly downregulated (Figure 9). In other words, due to the decrease in pyruvate dehydrogenase, the production of acetyl-CoA from pyruvate is decreased, which causes the increase in oxaloacetate and the decrease in citrate (Table 2). In conclusion, mitochondrial dysfunction along with a reduction in glycolysis and TCA cycle activity means that Caco-2 cells in the early stage of CDI experience a disturbance in the supply of the energy source needed to cope with stress.

**TABLE 2:** The metabolomic changes in Caco-2 cells during coculture with *C. difficile*. Metabolomic analysis was performed in quadruplicate.

**Name**	**Log_2_FC (*C. difficile*/control)**
Glutamate	−1.4062
Cysteine	−1.1766*
ATP	−5.3137***
Glutathione	−6.9352***
Glucose	−1.3254*
d-glucose-6-phosphate	−3.7082***
d-fructose-6-phosphate	−3.8165***
Citrate	−2.743***
Malate	−2.2069***
NADH	−2.0183*
Acetyl-CoA	−1.9625
Oxaloacetate	2.0266
Pyruvate	0.82646

*p < 0.05, **p < 0.01, ***p < 0.001.

## Conclusion

**FIGURE 10:**
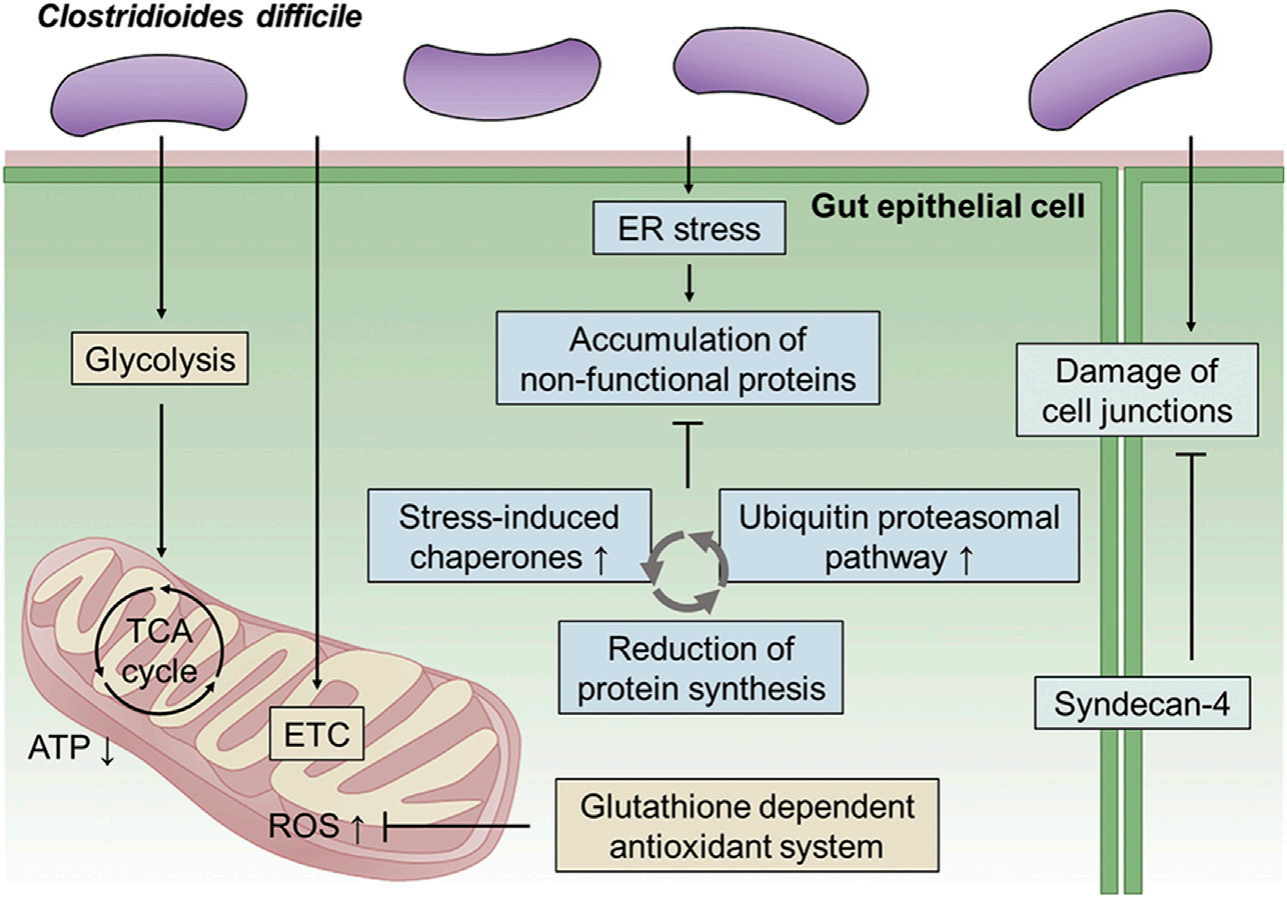
Schematic overview of metabolic regulation in Caco-2 cells cultured with *C. difficile*. First, *C. difficile* causes inhibition of energy metabolism, including glycolysis and the TCA cycle, along with downregulation of the electron transport chain in Caco-2 cells during the early stage of infection. Mitochondrial dysfunction leads to ROS production, and in response, Caco-2 cells upregulate the glutathione-dependent antioxidant system. Second, ER stress in Caco-2 cells caused by CDI causes the accumulation of nonfunctional proteins (unfolded and misfolded proteins). Caco-2 cells degrade nonfunctional proteins through the ubiquitin proteasomal pathway and reduce protein synthesis. Finally, *C. difficile* induces damage to cell junctions, including tight junctions and adherens junctions, in Caco-2 cells. In response, Caco-2 cells upregulate syndecan-4 to maintain intestinal epithelial barrier function. ETC, electron transfer chain; ER, endoplasmic reticulum.

In this study, a CDI model was constructed in an anaerobic environment using *C. difficile* and Caco-2 cells, which are human intestinal epithelial cells. An integrative multiomics analysis was used to investigate the physiological changes in intestinal epithelial cells caused by *C. difficile*. First, we validated our CDI model by confirming actin filament disaggregation and intestinal epithelial damage in the late stage (48 h) of the CDI model. Then, based on toxin accumulation and Caco-2 cell viability, proteomic and metabolomic analyses were performed at an early stage (12 h) of the CDI model. Even in the initial CDI model, most of the cell–cell junction proteins corresponding to the physical barrier of the cell were reduced, and in response, SDC4 upregulation was confirmed to maintain the intestinal epithelial barrier. Furthermore, it was discovered that Caco-2 cells upregulate the intracellular stress-induced chaperone complex to regulate irreversible protein aggregation in the presence of unfolded and misfolded proteins caused by *C. difficile* bacterial virulence factors. We next attempted to maintain intracellular protein homeostasis by decomposing abnormal proteins accumulated due to CDI through UPP. Additionally, activation of the antioxidant system that can protect cells from the generation of cytotoxic ROS was confirmed in the early stages of CDI. Finally, downregulation of the TCA cycle and glycolysis, which are components of energy metabolism, along with the decrease in ETC, showed that the intestinal epithelial cells were impaired in the supply of energy needed to cope with the stress caused by infection. In conclusion, in this study, an *in vitro* anaerobic-aerobic CDI model was constructed, and the biological mechanisms of changes in intestinal epithelial cells in the early stages of CDI could be detected at the molecular level through a multiomics approach (Figure 10). This system implemented like the actual intestinal CDI environment *in vivo*, and similar phenomena (i.e., damage the epithelial cell cytoskeleton, disruption of tight junction) were observed when compared with other CDI models using organoids. Also, the physiological and pathological processes of the host cells through interaction with *C. difficile* could be identified, and these results may provide a better understanding for identifying novel target mechanisms of CDI pathogenesis. Additionally, if our system is advanced through the introduction of primary cells and various gut components, it will be applicable as a platform technology for the discovery of next-generation CDI therapeutics and functional verification research.

## Data Availability

The datasets presented in this study can be found in online repositories. The names of the repository/repositories and accession number(s) can be found in the article/Supplementary Material.
