# Supplementary material for: Investigation of metabolic crosstalk between host and pathogenic Clostridioides difficile via multiomics approaches
Source: Front Bioeng Biotechnol. 2022 Sep 2;10:971739. doi: 10.3389/fbioe.2022.971739 (PMC9478559; doi:10.3389/fbioe.2022.971739)
Supplement: Supplementary file 1 [file DataSheet2.docx]

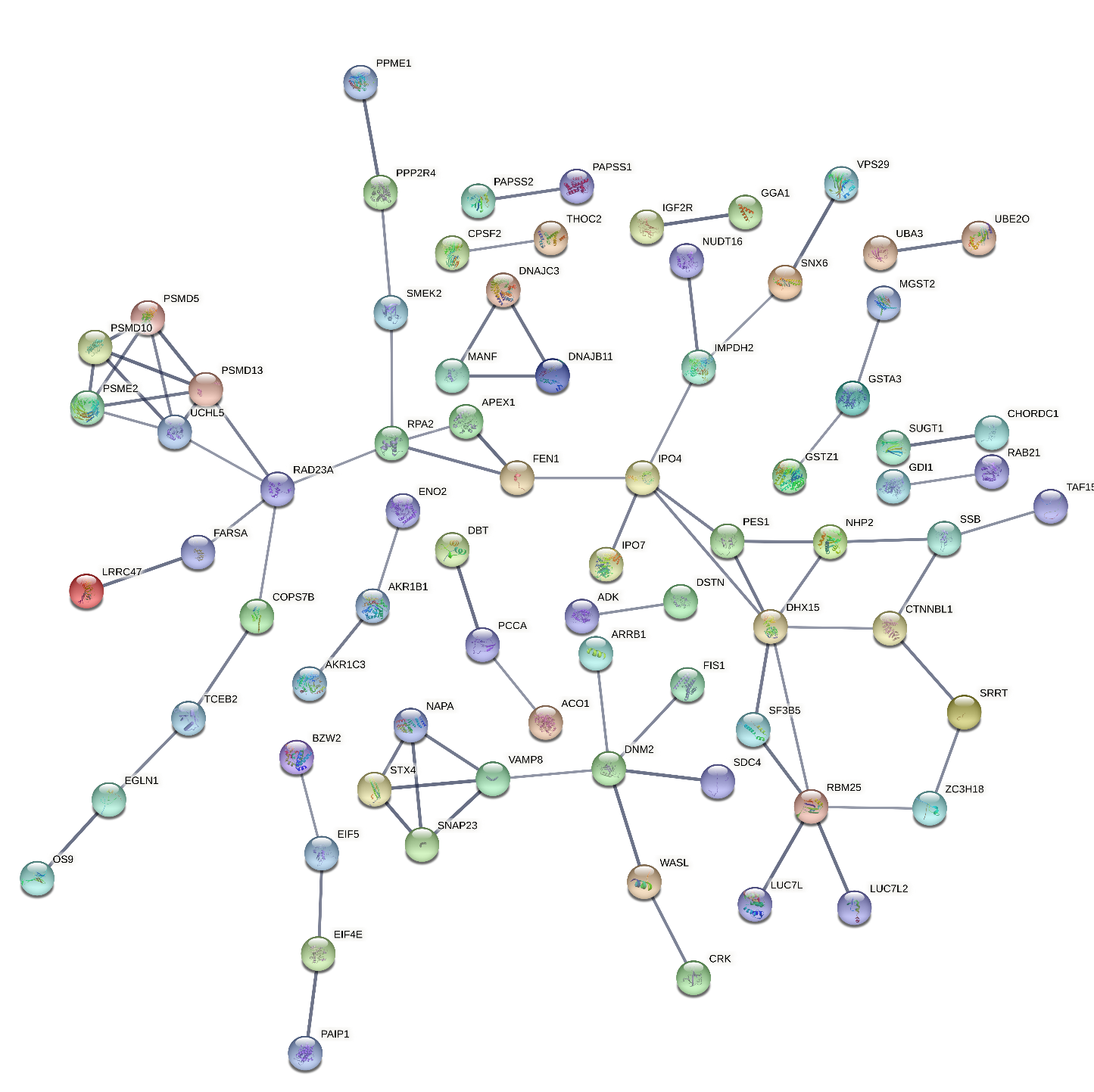
**Supplementary Figure S1.** STRING protein-protein interaction network of the 220 upregulated proteins. The minimum required interaction score was set at 0.7 and disconnected nodes were hidden in the network.


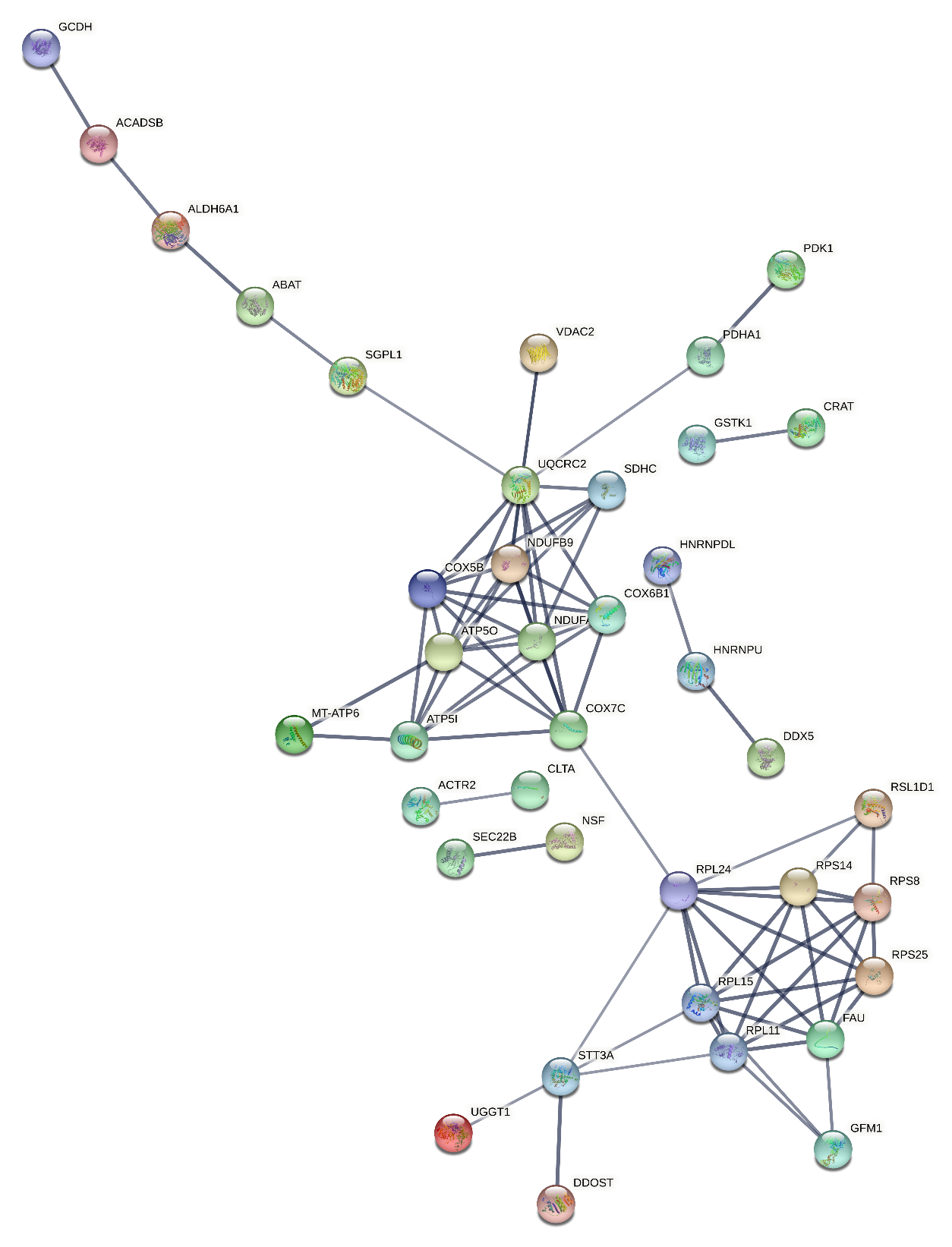
**Supplementary Figure S2.** STRING protein-protein interaction network of the 87 downregulated proteins. The minimum required interaction score was set at 0.7 and disconnected nodes were hidden in the network.

**Supplementary Figure S3.** Images of the stained porous membrane supports that Caco-2 cells were cultured in anaerobic chamber at 0, 12, 18, 24, 48 hr.


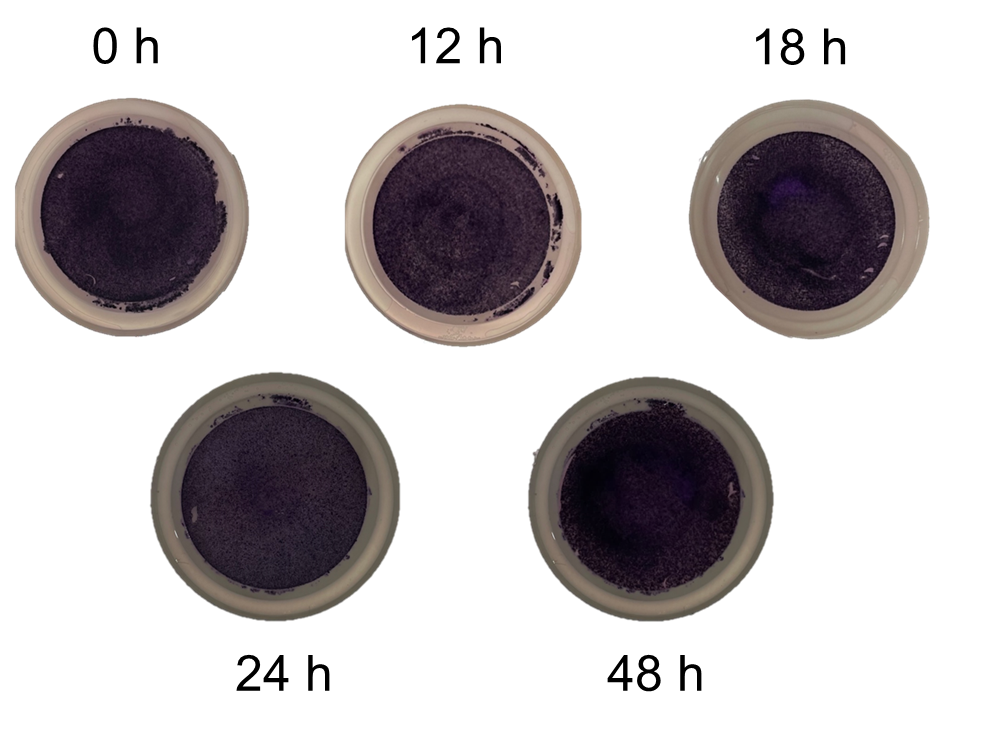


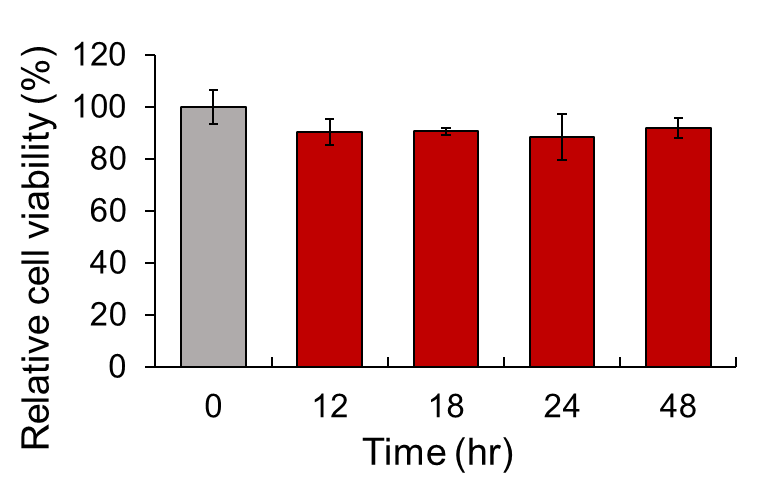
**Supplementary Figure S4.** Comparison of viabilities of Caco-2 cells cultured on the membrane supports in anaerobic chamber at 0, 12, 18, 24, 48 hr. The error bars represent the standard deviation of triplicate samples.

**Supplementary Figure S5.** Images of the stained porous membrane supports that Caco-2 cells according to coculture with *C. difficile* at 12, 24 and 48 hr related to 0 hr.


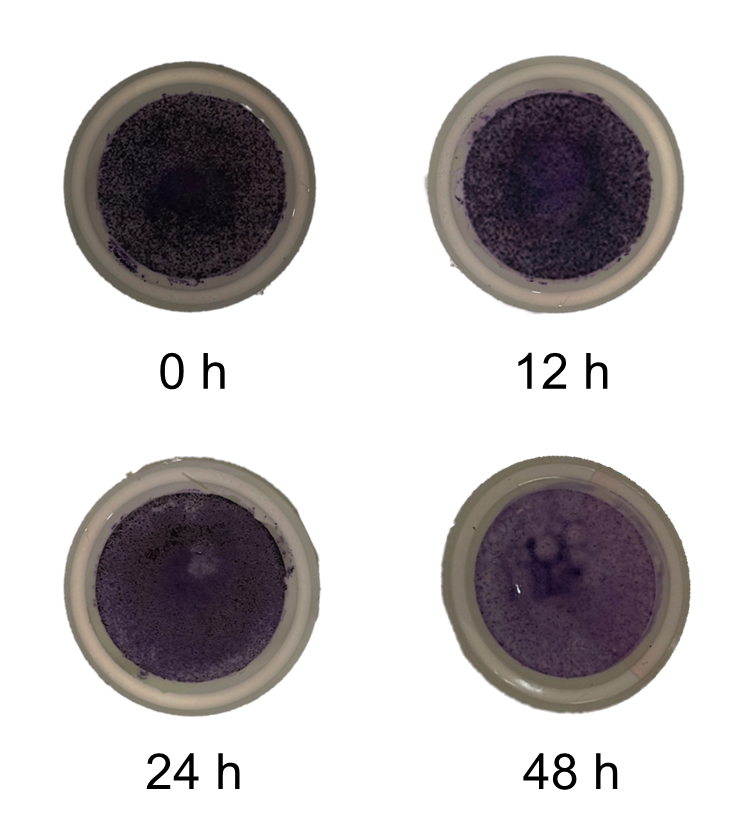


**Supplementary Table S1.** List of extracellular proteins secreted by *C. difficile* cultured with Caco-2 cells for 48 hr. riBAQ: relative label-free quantitation.

| Protein IDs | Geme names | iBAQ | riBAQ |
| --- | --- | --- | --- |
| A0A125V910 | slpA | 3268300000 | 18.78 |
| A0A125V1X2 | fliC | 2239100000 | 12.87 |
| A0A125VAJ3 | adhE_2 | 650920000 | 3.740 |
| A0A125V9Q4 | gapB | 640880000 | 3.683 |
| A0A125V4F4 | CDIF1296T_01244 | 472250000 | 2.714 |
| A0A125V9V7 | eno | 442720000 | 2.544 |
| A0A125V7N9 | CDIF1296T_02637 | 440420000 | 2.531 |
| A0A125VB78 | tpiA | 404300000 | 2.323 |
| A0A125V171 | tufA_2 | 357880000 | 2.056 |
| A0A125V1H9 | gluD | 254080000 | 1.460 |
| A0A125V5J7 | CDIF1296T_01544 | 243080000 | 1.397 |
| A0A125V3B0 | plfB | 240570000 | 1.382 |
| A0A125V9Z4 | pgk | 213810000 | 1.229 |
| A0A125YE00 | hupA | 212470000 | 1.221 |
| A0A125V405 | hbd | 201230000 | 1.156 |
| A0A125V8Y6 | CDIF1296T_02969 | 185640000 | 1.067 |
| A0A125V1E0 | proS | 178280000 | 1.024 |
| A0A125V2M9 | fba | 174090000 | 1.000 |
| A0A125V156 | rplL | 170530000 | 0.980 |
| A0A125V2C1 | etfB1 | 165490000 | 0.951 |
| A0A125V2B0 | acdB | 154310000 | 0.887 |
| A0A125V6U9 | fldX | 141950000 | 0.816 |
| A0A125V8V6 | nifJ | 135900000 | 0.781 |
| A0A125V408 | thlA1 | 125090000 | 0.719 |
| A0A125V455 | etfA2 | 123330000 | 0.709 |
| A0A125V485 | rplU | 118120000 | 0.679 |
| A0A125V3Z6 | rbr_1 | 114830000 | 0.660 |
| A0A125V2X1 | etfA1 | 111990000 | 0.644 |
| A0A125V5S3 | CDIF1296T_01778 | 110240000 | 0.633 |
| A0A125V4C1 | iscS2 | 98469000 | 0.566 |
| A0A125V8E4 | CDIF1296T_02230 | 93393000 | 0.537 |
| A0A125V8H4 | gabT | 93214000 | 0.536 |
| A0A125V3H6 | CDIF1296T_00976 | 89351000 | 0.513 |
| A0A125V242 | fliD | 82435000 | 0.474 |
| A0A125V1R3 | rplB | 81489000 | 0.468 |
| A0A125V807 | CDIF1296T_02523 | 81087000 | 0.466 |
| A0A125YDI5 | proC2 | 78772000 | 0.453 |
| A0A125V1X4 | flgL | 75554000 | 0.434 |
| A0A125YDI6 | pflD | 74556000 | 0.428 |
| A0A125V3Q8 | rbo | 74475000 | 0.428 |
| A0A125V178 | rpsH | 70347000 | 0.404 |
| A0A125V2B4 | ldhA | 69954000 | 0.402 |
| A0A125V4N6 | cspB | 68761000 | 0.395 |
| A0A125V837 | rpsT | 64137000 | 0.369 |
| A0A125V8U6 | appA | 61343000 | 0.352 |
| A0A125V4E7 | bcd2 | 59298000 | 0.341 |
| A0A125V2H3 | hadA | 58984000 | 0.339 |
| A0A125V3V6 | etfB2 | 57711000 | 0.332 |
| A0A125V187 | rpsC | 57265000 | 0.329 |
| A0A125V6A2 | cysD | 55925000 | 0.321 |
| A0A125V1B0 | rpsM | 55910000 | 0.321 |
| A0A125V2M2 | rplJ | 55272000 | 0.318 |
| A0A125YDR6 | pfkA | 54172000 | 0.311 |
| A0A125V189 | rplE | 53460000 | 0.307 |
| A0A125V536 | CDIF1296T_01039 | 53228000 | 0.306 |
| A0A125V8J2 | CDIF1296T_02755 | 53101000 | 0.305 |
| A0A125V542 | feoB1 | 51782000 | 0.298 |
| A0A125YED8 | CDIF1296T_03795 | 51184000 | 0.294 |
| A0A125V5B2 | CDIF1296T_01546 | 50707000 | 0.291 |
| A0A125V177 | rpsG | 50314000 | 0.289 |
| A0A125V4N4 | norV | 50147000 | 0.288 |
| A0A125V185 | rpmC | 50137000 | 0.288 |
| A0A125V5I5 | CDIF1296T_01799 | 49105000 | 0.282 |
| A0A125V7H2 | tkt'_1 | 48966000 | 0.281 |
| A0A125YED7 | rpsF | 47113000 | 0.271 |
| A0A125V8Q4 | glyA_1 | 46884000 | 0.269 |
| A0A125VA32 | ptsH | 45505000 | 0.261 |
| A0A125V9X8 | gpmI | 45220000 | 0.260 |
| A0A125YE91 | CDIF1296T_03732 | 43750000 | 0.251 |
| A0A125V1D6 | fusA | 41596000 | 0.239 |
| A0A125V1S6 | groES | 40270000 | 0.231 |
| A0A125YDK5 | clpP1 | 40103000 | 0.230 |
| A0A125V8X4 | CDIF1296T_02959 | 39390000 | 0.226 |
| A0A125V691 | CDIF1296T_02033 | 38596000 | 0.222 |
| A0A125V1B8 | rpsI | 37879000 | 0.218 |
| A0A125V1W1 | flgM | 37183000 | 0.214 |
| A0A125V1G0 | rpsE | 36654000 | 0.211 |
| A0A125V552 | CDIF1296T_01597 | 36288000 | 0.209 |
| A0A125YDX6 | atpD | 35561000 | 0.204 |
| A0A125V2Q8 | CDIF1296T_00752 | 35492000 | 0.204 |
| A0A125V8Y1 | CDIF1296T_02740 | 34718000 | 0.199 |
| A0A125V2L1 | CDIF1296T_00673 | 33935000 | 0.195 |
| A0A125V1A7 | rpsD | 33690000 | 0.194 |
| A0A125V752 | CDIF1296T_02298 | 32989000 | 0.190 |
| A0A125V2S4 | rplQ | 32870000 | 0.189 |
| A0A125V6S5 | tsf | 32406000 | 0.186 |
| A0A125YED3 | rplI | 32267000 | 0.185 |
| A0A125V4J3 | CDIF1296T_01346 | 31612000 | 0.182 |
| A0A125V7S0 | CDIF1296T_02506 | 31108000 | 0.179 |
| A0A125V5Q0 | ribH | 30784000 | 0.177 |
| A0A125YDR5 | pykF | 30674000 | 0.176 |
| A0A125V181 | serS1 | 30187000 | 0.173 |
| A0A125V7X6 | CDIF1296T_02569 | 29884000 | 0.172 |
| A0A125YEE3 | CDIF1296T_03800 | 29603000 | 0.170 |
| A0A125V633 | CDIF1296T_01730 | 29575000 | 0.170 |
| A0A125V872 | dnaK | 29484000 | 0.169 |
| A0A125V303 | rplT | 29178000 | 0.168 |
| A0A125V5K1 | gloA | 27887000 | 0.160 |
| A0A125YDX8 | atpA | 27261000 | 0.157 |
| A0A125V376 | pepD | 26931000 | 0.155 |
| A0A125V4V1 | rpsO | 26819000 | 0.154 |
| A0A125VA90 | tig | 25702000 | 0.148 |
| A0A125V157 | rplK | 24934000 | 0.143 |
| A0A125V2I1 | CDIF1296T_00666 | 24828000 | 0.143 |
| A0A125V2R4 | CDIF1296T_00747 | 24110000 | 0.139 |
| A0A125V7Y4 | CDIF1296T_02071 | 24101000 | 0.138 |
| A0A125V2B1 | hadB | 23918000 | 0.137 |
| A0A125V1K8 | rplM | 23788000 | 0.137 |
| A0A125V5J2 | CDIF1 | 23658000 | 0.136 |
| A0A125V155 | rplA | 22475000 | 0.129 |
| A0A125V4C4 | asp | 22069000 | 0.127 |
| A0A125V2P5 | rplN CDIF1296T_00149 | 22042000 | 0.127 |
| A0A125V2N3 | rplC CDIF1296T_00139 | 21869000 | 0.126 |
| A0A125V4X2 | CDIF1296T_01405 | 21670000 | 0.125 |
| A0A125V161 | rplD | 21650000 | 0.124 |
| A0A125V7I1 | rpsB | 21503000 | 0.124 |
| A0A125V4B5 | efp | 21226000 | 0.122 |
| A0A125V3F6 | CDIF1296T_00980 | 20708000 | 0.119 |
| A0A125V8V1 | ptsI | 20641000 | 0.119 |
| A0A125YDI9 | pgi | 19644000 | 0.113 |
| A0A125V1E7 | rplP | 19470000 | 0.112 |
| A0A125V1C3 | metE | 19452000 | 0.112 |
| A0A125V7C6 | guaB | 19421000 | 0.112 |
| A0A125V184 | rplO | 19332000 | 0.111 |
| A0A125V4Q9 | rpmF | 18296000 | 0.105 |
| A0A125V8D6 | leuS | 18231000 | 0.105 |
| A0A125YDF2 | prdB_1 | 17772000 | 0.102 |
| A0A125V8B5 | CDIF1296T_02625 | 17234000 | 0.099 |
| A0A125V6N4 | CDIF1296T_02093 | 17112000 | 0.098 |
| A0A125VAT4 | prs | 16953000 | 0.097 |
| A0A125V2M3 | CDIF1296T_00704 | 15932000 | 0.092 |
| A0A125V167 | rpsS | 15469000 | 0.089 |
| A0A125V795 | CDIF1296T_02483 | 15463000 | 0.089 |
| A0A125V1R8 | CDIF1296T_00304 | 15271000 | 0.088 |
| A0A125V5L7 | trxA1 | 14883000 | 0.086 |
| A0A125V8N7 | CDIF1296T_02906 | 14774000 | 0.085 |
| A0A125V4D9 | rplS | 14547000 | 0.084 |
| A0A125V463 | rnfB | 14481000 | 0.083 |
| A0A125V193 | rplR | 14401000 | 0.083 |
| A0A125V751 | tal1 | 13672000 | 0.079 |
| A0A125V1C1 | CDIF1296T_00230 | 13032000 | 0.075 |
| A0A125V7B3 | CDIF1296T_02503 | 12816000 | 0.074 |
| A0A125YDY6 | rpiB2 | 12742000 | 0.073 |
| A0A125V5W0 | rpsP | 12732000 | 0.073 |
| A0A125V467 | ackA | 12581000 | 0.072 |
| A0A125V436 | CDIF1296T_01185 | 12418000 | 0.071 |
| A0A125V844 | CDIF1296T_02814 | 12395000 | 0.071 |
| A0A125V1L1 | groEL | 12359000 | 0.071 |
| A0A125V6I5 | metQ | 12182000 | 0.070 |
| A0A125V8B9 | rpe | 11814000 | 0.068 |
| A0A125VAX8 | greA | 11727000 | 0.067 |
| A0A125VAG7 | CDIF1296T_03506 | 11369000 | 0.065 |
| A0A125YDX3 | alr | 10785000 | 0.062 |
| A0A125V206 | flgE | 10452000 | 0.060 |
| A0A125V5J6 | CDIF1296T_01715 | 10363000 | 0.060 |
| A0A125V1B5 | glmS | 10282000 | 0.059 |
| A0A125V1Z3 | hpf | 10073000 | 0.058 |
| A0A125V7J7 | tkt_1 | 10047000 | 0.058 |
| A0A125V1L3 | guaA | 9872900 | 0.057 |
| A0A125YEL3 | CDIF1296T_phi007 | 9569200 | 0.055 |
| A0A125V6H4 | accB | 23807000 | 0.137 |
| A0A125V1J3 | CDIF1296T_00299 | 9562000 | 0.055 |
| A0A125V1B7 | CDIF1296T_00233 | 9438700 | 0.054 |
| A0A125YE23 | ksgA | 9431400 | 0.054 |
| A0A125V281 | CDIF1296T_00538 | 9028800 | 0.052 |
| A0A125V5F0 | CDIF1296T_01686 | 8975600 | 0.052 |
| A0A125V510 | CDIF1296T_01353 | 8687500 | 0.050 |
| A0A125V3U9 | hadC | 8683100 | 0.050 |
| A0A125V3P9 | CDIF1296T_01047 | 8645500 | 0.050 |
| A0A125V2A0 | ppaC | 7919700 | 0.046 |
| A0A125V5C6 | CDIF1296T_01733 | 7826100 | 0.045 |
| A0A125V1S4 | rex | 7814800 | 0.045 |
| A0A125V430 | CDIF1296T_01209 | 7669700 | 0.044 |
| A0A125V9U5 | ptsG | 7423100 | 0.043 |
| A0A125V6I6 | CDIF1296T_01986 | 7060200 | 0.041 |
| A0A125V421 | oppA | 6864800 | 0.039 |
| A0A158SIG9 | CDIF1296T_phi112 | 6810300 | 0.039 |
| A0A125V158 | rpsJ | 6772200 | 0.039 |
| A0A125V7Y9 | gatB | 6305100 | 0.036 |
| A0A125V7Z1 | CDIF1296T_02754 | 6103600 | 0.035 |
| A0A125V537 | CDIF1296T_01595 | 5930000 | 0.034 |
| A0A125V2U4 | thrS | 5916000 | 0.034 |
| A0A125V1G9 | rplV | 5820400 | 0.033 |
| A0A125V8Q0 | CDIF1296T_02922 | 5770300 | 0.033 |
| A0A125V515 | CDIF1296T_01609 | 5719000 | 0.033 |
| A0A125V647 | CDIF1296T_01983 | 5717800 | 0.033 |
| A0A125V1A0 | rpoA | 5573900 | 0.032 |
| A0A125V173 | CDIF1296T_00131 | 5443900 | 0.031 |
| A0A125V8K5 | CDIF1296T_02280 | 5322200 | 0.031 |
| A0A125V4A7 | fabK | 4952000 | 0.028 |
| A0A125V4E8 | codY | 4880100 | 0.028 |
| A0A125V9M4 | CDIF1296T_03251 | 4864200 | 0.028 |
| A0A125V960 | CDIF1296T_02929 | 4679300 | 0.027 |
| A0A125V258 | purC | 4651000 | 0.027 |
| A0A125YE63 | CDIF1296T_03696 | 4542700 | 0.026 |
| A0A125VA25 | CDIF1296T_03151 | 4402300 | 0.025 |
| A0A125V278 | htpG | 4372700 | 0.025 |
| A0A125V2G1 | CDIF1296T_00630 | 4369800 | 0.025 |
| A0A125V4G6 | fur | 4338000 | 0.025 |
| A0A125V3L4 | CDIF1296T_01075 | 4179800 | 0.024 |
| A0A125V438 | mreB2 | 4075600 | 0.023 |
| A0A125V877 | tdcB | 4009900 | 0.023 |
| A0A125V8G2 | CDIF1296T_02747 | 3970700 | 0.023 |
| A0A125V2R7 | CDIF1296T_00748 | 3871000 | 0.022 |
| A0A125V1V1 | purH | 3851400 | 0.022 |
| A0A125V5A0 | aspB | 3762300 | 0.022 |
| A0A125V550 | metN | 3642300 | 0.021 |
| A0A125V3T9 | crt2 | 3476800 | 0.020 |
| A0A125YDY0 | atpF | 3447900 | 0.020 |
| A0A125V1D0 | CDIF1296T_00232 | 3321300 | 0.019 |
| A0A125YE85 | luxS | 3107900 | 0.018 |
| A0A125V7U1 | iorB | 3031800 | 0.017 |
| A0A125V529 | CDIF1296T_01539 | 3030300 | 0.017 |
| A0A125V1V0 | luxS | 2925000 | 0.017 |
| A0A125V799 | iorB | 2917100 | 0.017 |
| A0A125V4T9 | CDIF1296T_01484 | 2834800 | 0.016 |
| A0A125V1Q3 | rpoB | 2819300 | 0.016 |
| A0A125V7Q9 | CDIF1296T_02659 | 2794300 | 0.016 |
| A0A125V6H7 | CDIF1296T_02060 | 2632500 | 0.015 |
| A0A125V4M9 | comR | 2573400 | 0.015 |
| A0A125V9D2 | CDIF1296T_03054 | 151130000 | 0.868 |
| A0A125V7A4 | asnC | 2535400 | 0.015 |
| A0A125V3R3 | leuD | 2514300 | 0.014 |
| A0A125V705 | hfQ | 2350600 | 0.014 |
| A0A125V577 | leuC | 2339700 | 0.013 |
| A0A125V1W0 | flgK | 2313100 | 0.013 |
| A0A125V223 | purG | 2307200 | 0.013 |
| A0A125V9F8 | cwp66 | 2292300 | 0.013 |
| A0A125V5C0 | cysM | 2203000 | 0.013 |
| A0A125V3I2 | CDIF1296T_01011 | 2061800 | 0.012 |
| A0A125V3J2 | CDIF1296T_01008 | 2039500 | 0.012 |
| A0A125V356 | fhs | 1979700 | 0.011 |
| A0A125V7T7 | glyS | 1927600 | 0.011 |
| A0A125V722 | hcp | 1838100 | 0.011 |
| A0A125V2V1 | glmM | 1833900 | 0.011 |
| A0A125V9S6 | ftsZ | 1714700 | 0.010 |
| A0A125V596 | CDIF1296T_01703 | 1678300 | 0.010 |
| A0A125V6C0 | CDIF1296T_02122 | 1635400 | 0.009 |
| A0A125YE35 | metG | 1576200 | 0.009 |
| A0A125V559 | tyrR | 1462300 | 0.008 |
| A0A125V8U8 | CDIF1296T_02967 | 1443200 | 0.008 |
| A0A125YDF3 | prdB_2 | 1392800 | 0.008 |
| A0A125V8U9 | CDIF1296T_02905 | 1344600 | 0.008 |
| A0A125YDH5 | pepF | 1312300 | 0.008 |
| A0A125V8W9 | CDIF1296T_03124 | 1304500 | 0.007 |
| A0A125V165 | rpoC | 1265400 | 0.007 |
| A0A125V1V2 | purF | 1242900 | 0.007 |
| A0A125V7Z0 | CDIF1296T_02603 | 1221400 | 0.007 |
| A0A125V5Q1 | CDIF1296T_01862 | 1126300 | 0.006 |
| A0A125V7L3 | fruK | 988430 | 0.006 |
| A0A125V852 | CDIF1296T_02677 | 900670 | 0.005 |
| A0A125V4K4 | dapG | 859950 | 0.005 |
| A0A125V5L2 | pgm1 | 832400 | 0.005 |
| A0A125V3M8 | CDIF1296T_01086 | 796580 | 0.005 |
| A0A125V478 | fabF | 784900 | 0.005 |
| A0A125VA31 | prdF | 741360 | 0.004 |
| A0A125YEE2 | CDIF1296T_03799 | 727880 | 0.004 |
| A0A125V7G4 | CDIF1296T_02370 | 493810 | 0.003 |
| A0A125V4V9 | CDIF1296T_01523 | 472900 | 0.003 |
| A0A125V1B1 | buk | 463090 | 0.003 |
| A0A125YDW8 | CDIF1296T_03574 | 411800 | 0.002 |
| A0A125V4P0 | recA | 380460 | 0.002 |
| A0A125V434 | CDIF1296T_01131 | 348400 | 0.002 |
| A0A125V5D8 | CDIF1296T_01691 | 321380 | 0.002 |
| A0A125V7T9 | grdD | 318060 | 0.002 |
| A0A125V147 | proS | 194720 | 0.001 |

**Supplementary Table S3.** List of identified proteins belonging to the ribosome subunit. Proteomic analysis was performed in triplicates. *p < 0.05, **p < 0.01, ***p < 0.001.

| UniProt IDs | Gene names | Protein names | Log_2_FC (*C. difficile*/control) |
| --- | --- | --- | --- |
| 1. 40S ribosomal proteins | | | |
| P08865 | RPSA | 40S ribosomal protein SA | -0.353^*^ |
| P62241 | RPS8 | 40S ribosomal protein S8 | -1.072^*^ |
| P62753 | RPS6 | 40S ribosomal protein S6 | -0.944^*^ |
| P62851 | RPS25 | 40S ribosomal protein S25 | -1.028^*^ |
| P61247 | RPS3A | 40S ribosomal protein S3a | -0.930^**^ |
| P62263 | RPS14 | 40S ribosomal protein S14 | -1.023^**^ |
| P39019 | RPS19 | 40S ribosomal protein S19 | -0.905^**^ |
| P62280 | RPS11 | 40S ribosomal protein S11 | -0.724^*^ |
| P25398 | RPS12 | 40S ribosomal protein S12 | -0.629^*^ |
|  |  |  |  |
| 2. 60S ribosomal proteins | | | |
| P62913 | RPL11 | 60S ribosomal protein L11 | -1.208^*^ |
| P62424 | RPL7A | 60S ribosomal protein L7a | -0.804^*^ |
| P83731 | RPL24 | 60S ribosomal protein L24 | -1.435^*^ |
| P61313 | RPL15 | 60S ribosomal protein L15 | -1.194^*^ |
| P62829 | RPL23 | 60S ribosomal protein L23 | -0.887^**^ |
| P30050 | RPL12 | 60S ribosomal protein L12 | -0.739^*^ |
| P05387 | RPLP2 | 60S acidic ribosomal protein P2 | -0.751^***^ |
| P05388 | RPLP0 | 60S acidic ribosomal protein P0 | -0.718^*^ |

**Supplementary Figure S6.** Comparison of metabolomic results of Caco‐2 cells by coculture with *C. difficile* at 12 hr according to monoculture. (A) Volcano plot for comparison of metabolomic difference according to coculture with *C. difficile* at 12 hr. Volcano plots indicate the upregulated metabolites of Caco‐2 cells by coculture with *C. difficile* in red circles and downregulated metabolites in blue circles. Indications are metabolites discussed in this study. (B) Principal component analysis (PCA) plot for comparison of metabolomic difference according to coculture with *C. difficile* at 12 hr. Control means monoculture of Caco‐2 cells. Metabolomic analysis was performed in quadruplicates.


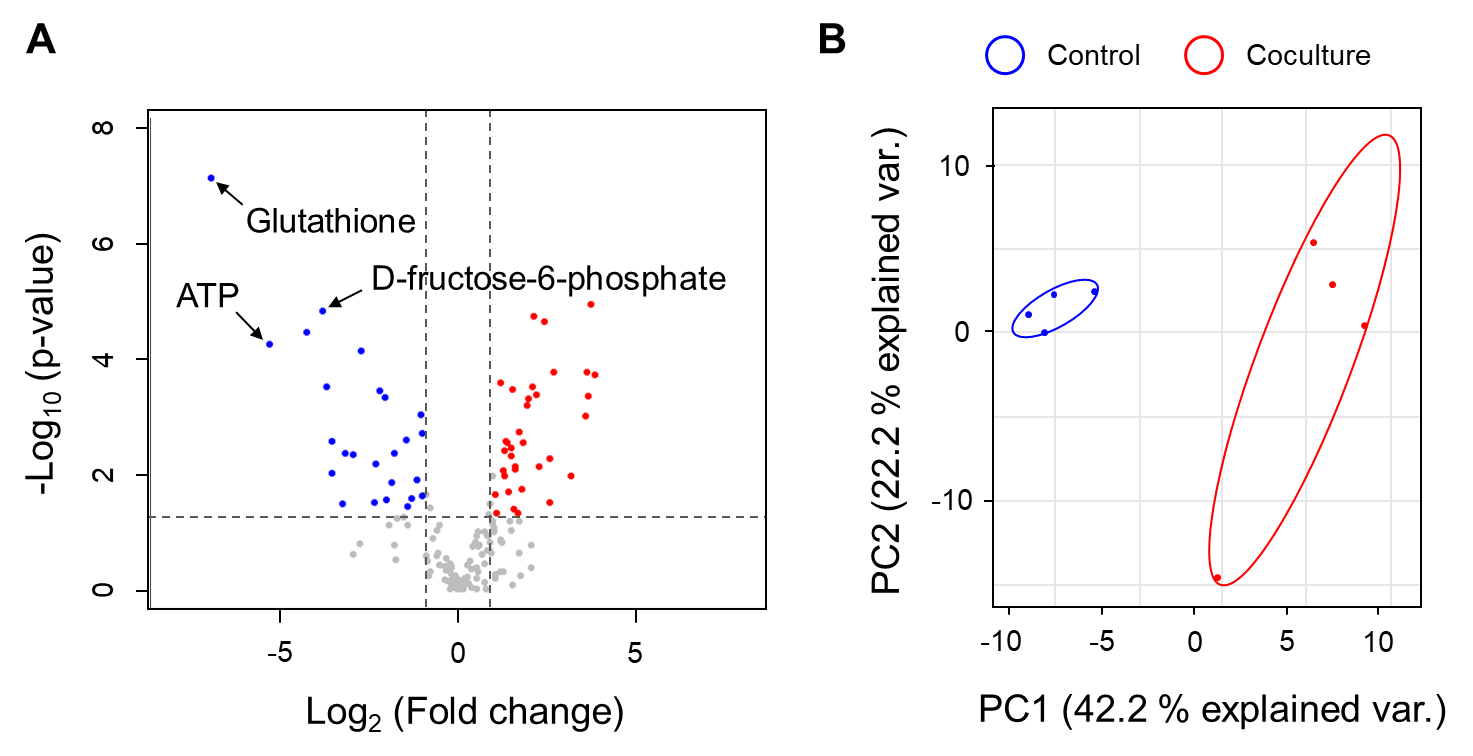


**Supplementary Table S4.** Metabolomic changes of Caco-2 cells by coculture with *C. difficile*. Metabolomic analysis was performed in quadruplicates. The FDR-corrected *p*-values were provided using the Metaboanalyst 5.0.

| Metabolite names | Fold change (Log2) | p-value (-Log10) |
| --- | --- | --- |
| Glutathione | -6.9352 | 7.1205 |
| Adenosine triphosphate | -5.3137 | 4.2553 |
| Guanosine triphosphate | -4.2386 | 4.466 |
| D-fructose-6-phosphate | -3.8165 | 4.8351 |
| D-glucose-6-phosphate | -3.7082 | 3.5052 |
| Cytidine triphosphate | -3.5458 | 2.5669 |
| Aspartic acid | -3.5334 | 2.0227 |
| Deoxyguanosine monophosphate | -3.243 | 1.478 |
| Dihydrofolate | -3.169 | 2.3708 |
| Threonine | -2.9475 | 2.333 |
| Cysteinesulfinilate | -2.9332 | 0.61894 |
| Deoxyguanosine | -2.7676 | 0.79839 |
| Citric acid | -2.743 | 4.139 |
| Proline | -2.3669 | 1.517 |
| Homocysteic acid | -2.2992 | 2.1886 |
| Malic acid | -2.2069 | 3.4438 |
| UDP-glucuronic acid | -2.0579 | 3.3253 |
| NADH | -2.0183 | 1.5463 |
| Acetyl-CoA | -1.9625 | 1.1124 |
| Pyridinedicarboxylic acid | -1.88 | 1.8679 |
| Homocysteine | -1.8098 | 2.3556 |
| Thymidine diphosphate | -1.7883 | 0.7831 |
| Uridine monophosphate | -1.7463 | 0.5158 |
| Alanine | -1.7292 | 1.2283 |
| Glutamine | -1.5496 | 1.2542 |
| Erythrose phosphate | -1.4686 | 2.604 |
| UDP-acetylglucosamine | -1.4295 | 1.4463 |
| Glutamic acid | -1.4062 | 1.1216 |
| Glucose | -1.3254 | 1.5865 |
| Cysteine | -1.1766 | 1.9067 |
| Guanidineacetic acid | -1.04 | 3.0313 |
| Valine | -1.0132 | 2.7031 |
| Nicotinamide | -1.0064 | 1.622 |
| Cytidine diphosphate | -0.95395 | 1.6454 |
| Taurodeoxycholate | -0.89585 | 0.59657 |
| Isoleucine | -0.88885 | 1.6564 |
| NAD | -0.86923 | 0.49354 |
| Cysteic acid | -0.81426 | 0.23826 |
| Hydroxy proline | -0.77575 | 1.4243 |
| Isobutyryl-CoA | -0.77553 | 0.30439 |
| Spermidine | -0.71426 | 0.90127 |
| Allantoin | -0.5967 | 1.0298 |
| Choline | -0.58479 | 0.59696 |
| Cytidine monophosphate | -0.58129 | 0.64206 |
| Phenylalanine | -0.53984 | 0.43192 |
| Tyrosine | -0.53304 | 1.117 |
| CoA | -0.39798 | 0.18214 |
| Deoxycytidine monophosphate | -0.3892 | 0.41613 |
| Indoleacrylic acid | -0.3541 | 0.33309 |
| Xanthosine monophosphate | -0.33094 | 0.54031 |
| Methionine | -0.31885 | 0.39519 |
| Tryptophan | -0.30877 | 0.32715 |
| Aminobutyric acid | -0.2876 | 0.15086 |
| Maltotriose | -0.24052 | 0.017164 |
| Aconitic acid | -0.22199 | 0.45663 |
| Adenosine diphosphate | -0.21016 | 0.41684 |
| Adenosylhomocysteine | -0.19261 | 0.27217 |
| Deoxyguanosine triphosphate | -0.18777 | 0.38247 |
| Deoxyadenosine | -0.17861 | 0.10791 |
| Asparagine | -0.16465 | 0.22216 |
| Deoxyadenosine monophosphate | -0.14974 | 0.09731 |
| Histidine | -0.11309 | 0.25083 |
| Hydroxyectoine | -0.11203 | 0.25162 |
| Acetyl-serine | -0.085875 | 0.042296 |
| Pyridoxamine | -0.072973 | 0.11268 |
| Chenodeoxycholic acid | -0.067335 | 0.23502 |
| Deoxycytidine triphosphate | -0.054646 | 0.20212 |
| Phosphoenolpyruvic acid | -0.047969 | 0.15029 |
| Hemin | -0.029348 | 0.024691 |
| Propionyl-CoA | -0.017423 | 0.10745 |
| Pyrrolidinone | -0.015261 | 0.0619 |
| Fumaric Acid | -0.0070825 | 0.025553 |
| Folic acid | 0.031334 | 0.006054 |
| Cyanocobalamin | 0.082295 | 0.14287 |
| Aminoadipic acid | 0.13228 | 0.050267 |
| Methyladipic acid | 0.14626 | 0.004802 |
| Citrulline | 0.14986 | 0.050968 |
| Arginine | 0.18107 | 0.1092 |
| Deoxyguanosine diphosphate | 0.19059 | 0.20422 |
| Galacturonic acid | 0.21479 | 0.1282 |
| Guanosine monophosphate | 0.23967 | 0.21438 |
| Diphosphoglyceric acid | 0.27159 | 0.43348 |
| Deoxyuridine | 0.28666 | 0.11376 |
| Dimethylglycine | 0.3412 | 0.50842 |
| Phosphogluconic acid | 0.34272 | 0.50681 |
| Taurocholic acid | 0.34926 | 0.048073 |
| Uridine | 0.37018 | 0.35308 |
| Furoic acid | 0.40457 | 0.74653 |
| Trehalose | 0.48684 | 0.81538 |
| Cystathionine | 0.49341 | 0.00080778 |
| Dihydroxybenzoic acid | 0.51601 | 0.20115 |
| Pyridoxine | 0.51835 | 0.94254 |
| Guanosine diphosphate | 0.52039 | 0.38985 |
| Gluconate | 0.52063 | 0.01175 |
| Lactic acid | 0.54577 | 1.0096 |
| Isopropylmalic acid | 0.55492 | 0.75906 |
| Dihydroxyacetone phosphate | 0.59226 | 0.76387 |
| Betaine | 0.6493 | 0.6039 |
| Serine | 0.72188 | 0.1259 |
| Lactulose | 0.73437 | 0.45824 |
| Succinic acid | 0.73876 | 1.0118 |
| Ribose phosphate | 0.77233 | 0.019263 |
| Flavin adenine dinucleotide | 0.82391 | 0.6806 |
| Pyruvic acid | 0.82646 | 0.9352 |
| Tetrahydrofolic acid | 0.85269 | 1.2945 |
| Phosphoglyceric acid | 0.85775 | 0.93709 |
| Thymidine monophosphate | 0.88285 | 1.4874 |
| Pyridoxic acid | 0.88358 | 0.68893 |
| Inosine monophosphate | 0.8986 | 0.82531 |
| D-Biotin | 0.92049 | 0.64478 |
| Maleic acid | 0.94757 | 1.1824 |
| Uridine triphosphate | 0.95747 | 1.9659 |
| Dihydrouracil | 0.97152 | 1.1507 |
| Citramalic acid | 0.98964 | 0.99995 |
| Anthranilamide | 1.0038 | 1.0799 |
| Uridine diphosphate | 1.0222 | 1.647 |
| Glucosamine phosphate | 1.0391 | 0.19812 |
| Butyryl-CoA | 1.0539 | 0.27919 |
| Taurochenodeoxycholic acid | 1.0549 | 1.3252 |
| Homoserine | 1.1659 | 0.32069 |
| Serotonin | 1.1716 | 0.86232 |
| Inosine | 1.1766 | 0.85199 |
| Deoxycytidine | 1.192 | 3.5934 |
| Glyceric acid | 1.2149 | 0.81789 |
| Uracil | 1.2565 | 2.0713 |
| Molybdate | 1.2708 | 0.3161 |
| Thymidine | 1.2902 | 1.981 |
| Thymine | 1.3024 | 2.4188 |
| Indole-propionic acid | 1.3442 | 2.5812 |
| Taurolithocholic acid | 1.3708 | 2.5464 |
| Acetyl glucosamine | 1.4217 | 1.7014 |
| Shikimic acid | 1.4508 | 1.185 |
| Hydroxycoumarin | 1.4904 | 2.4641 |
| Deoxyuridine monophosphate | 1.4917 | 2.316 |
| Deoxyadenosine triphosphate | 1.4998 | 1.0169 |
| Hypoxanthine | 1.5171 | 3.475 |
| Ketoglutaric acid | 1.5227 | 0.091168 |
| Orotic acid | 1.5484 | 1.4076 |
| Hydroxyanthranilic acid | 1.5764 | 2.1283 |
| Nicotinamide mononucleotide | 1.5843 | 2.0938 |
| Acetyl phosphate | 1.6842 | 1.3371 |
| Indole-carboxaldehyde | 1.7042 | 1.1881 |
| Menadione | 1.7135 | 0.62801 |
| Deoxyinosine | 1.7227 | 2.7221 |
| Deoxycytidine diphosphate | 1.7522 | 0.24834 |
| Lysine | 1.7819 | 1.7442 |
| Oxobutyric acid | 1.8148 | 2.5551 |
| Hydroxyphenylacetic acid | 1.9287 | 3.1959 |
| Nicotinic acid | 1.9789 | 3.3171 |
| Oxaloacetic acid | 2.0266 | 0.37428 |
| Adenosine monophosphate | 2.0373 | 0.77286 |
| Hydroxymethylbenzoic acid | 2.088 | 3.5215 |
| Xanthosine | 2.0976 | 4.7338 |
| Guanine | 2.1718 | 3.3662 |
| Formyl-methionine | 2.2587 | 2.1277 |
| Guanosine | 2.41 | 4.633 |
| Hydroxyphenylpyruvic acid | 2.5617 | 2.2813 |
| Adenine | 2.5749 | 1.5092 |
| Dihydroorotic acid | 2.6716 | 3.774 |
| Adenosine | 3.1588 | 1.9621 |
| Phenylpyruvate | 3.5677 | 3.0009 |
| Azacytidine | 3.6115 | 3.7786 |
| Cytosine | 3.6535 | 3.3426 |
| Citraconic acid | 3.7296 | 4.9412 |
| Cytidine | 3.817 | 3.7113 |
